# Amphiphilicity Controls Nanostructure in Surfactant-Free Ionic Liquid Microemulsions

**DOI:** 10.1021/jacs.6c13294

**Published:** 2026-09-07

**Authors:** Haihui Joy Jiang, Silvia Imberti, Kathleen Wood, Rob Atkin, Gregory G. Warr

**Affiliations:** † School of Chemistry and Sydney Nano Institute, 4334 The University of Sydney, NSW 2006, Australia; ‡ STFC, Rutherford Appleton Laboratory, Didcot, Oxfordshire OX11 OQX, U.K.; § Australian Centre for Neutron Scattering, Australian Nuclear Science and Technology Organization, New Illawarra Road, Lucas Heights 2234, New South Wales, Australia; ∥ School of Molecular Sciences, The University of Western Australia, Perth WA 6009, Australia

## Abstract

Traditional microemulsions are ubiquitous nanostructured liquids stabilized by high surfactant concentrations that can be a significant drawback. Here, we show that surfactant-free microemulsions can be formulated when amphiphilicity is incorporated directly into a protic ionic liquid solvent. Using combined high-resolution X-ray and neutron scattering with isotopic labeling, we show that an amphiphilic ionic liquid, propylammonium nitrate, functions both as a polar solvent and hydrotrope, generating polar–apolar nanostructured domains without a conventional surfactant monolayer. In contrast, a non-amphiphilic ionic liquid, ethanolammonium nitrate, simply acts as a polar solvent, driving solvophobic segregation in purely non-aqueous mixtures. The balance between Coulomb interactions, hydrogen bonding, and amphiphilicity determines domain structure and phase coexistence. These results show that cation amphiphilicity is the molecular parameter that controls the nanostructure and phase behavior in surfactant-free microemulsions. This behavior is expected to apply to systems in which amphiphilicity is embedded within the solvent.

## Graphical abstract

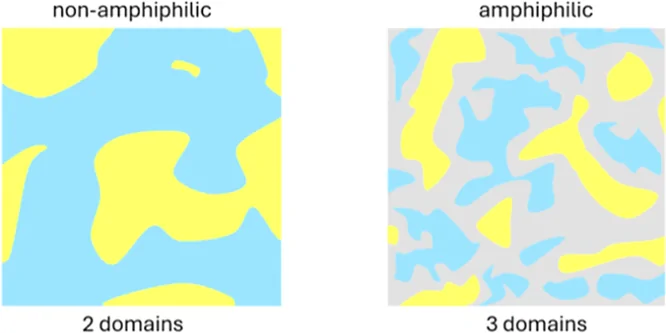

## Introduction

A microemulsion is traditionally defined as “a dispersion made of water, oil, and surfactant(s) that is an isotropic and thermodynamically stable system with dispersed domain diameter varying approximately from 1 to 100 nm”.^1^ The coexistence of polar and apolar domains within a single liquid phase underpins their effectiveness for applications as nano-reactors, as media for nano-material templating, in pharmaceutical delivery, and as food additives.^2–6^ However, high surfactant concentrations present ongoing challenges including separation of residual contaminants from product, toxicity, cost, and environmental impact. This has motivated strategies seeking to reduce conventional surfactant content^7^ or develop alternative formulations^8,9^ that exhibit an amphiphilic nanostructure.

Ultra-flexible or surfactant-free microemulsions are thermodynamically stable mixtures of polar and apolar components in which a hydrotrope or a cosurfactant mediates domain formation^10^ without the need for a traditional compact surfactant monolayer.^11–13^ These are exemplified by water–ethanol–octanol, in which weakly amphiphilic ethanol accumulates at the water–octanol interface^9,13,14^ Mixtures such as these have shown enhanced solubilization capacity, preservation of enzymatic activity, and long-lasting effects in olfaction^15–20^ and have been explored as media for liquid–liquid extraction.

Ionic liquids (ILs) offer a fundamentally different approach to microemulsion design. Many pure ILs contain amphiphilic ions, i.e., those that comprise distinct polar, charged groups bonded to non-polar (usually alkyl) moieties. This induces the nanostructure in the bulk due to attractions between ions creating polar domains that solvophobically exclude apolar groups, which results in the formation of polar and apolar domains whose size and spatial arrangement depend on ion structure.^21,22^ This creates the opportunity to incorporate amphiphilicity directly into the polar solvent rather than by addition of a separate self-assembling component such as a conventional surfactant. While their negligible vapor pressure, chemical tunability, and, in many cases, green chemical characteristics have led to their incorporation into conventional microemulsions to replace oil, water, and surfactant components,^23–30^ their potential to intrinsically generate and control the nanostructure through their own molecular architecture remains largely unexplored.

Propylammonium nitrate (PAN) is a model amphiphilic IL whose cation contains distinct charged groups and propyl chains that give rise to both polar–apolar nanostructure and interfacial activity. Pure PAN forms a sponge-like liquid comprising bicontinuous polar and apolar domains^31^ and forms an oriented film at a free surface.^32,33^ Ethanolammonium nitrate (EtAN), the hydroxyl-terminated analogue of PAN, is a well-studied example of a polar, non-amphiphilic ionic liquid that, like water, forms a dense, three-dimensional H-bond network,^34–37^ has a high surface tension with negligible surface enrichment in aqueous mixtures,^38^ and only weakly orients at free surfaces.^39^ The two cations are of similar size and charge, differing principally in whether the chain terminates in a methyl or a hydroxyl group, altering H-bonding capacity and polarity, but previous work has shown that amphiphilicity dominates their bulk and interfacial structure and behavior.^22,31–34,38–40^ This suggests that ionic liquid amphiphilicity may act as a molecular control parameter for miscibility, domain structure, and phase equilibria in surfactant-free microemulsions.

Here, we test this hypothesis by investigating the ternary mixtures of protic ionic liquids with polar and apolar components. PAN is used as both a polar solvent and hydrotrope (co-solvent), while EtAN acts as a non-amphiphilic, polar reference solvent. Molecular organization is resolved across multiple length scales using X-ray and neutron scattering with multiple isotopic labeling (see the Supporting Information). By correlating intermolecular interactions with liquid nanostructure and phase behavior, we show how cation amphiphilicity controls the polar–apolar nanostructure and coexisting phase compositions in surfactant-free microemulsions.

## Results

### Amphiphilic Ionic Liquid as a Hydrotrope

The role of amphiphilicity is first examined in water-alcohol systems in which an amphiphilic ionic liquid, PAN, replaces a conventional hydrotrope. The water–PAN–octanol ternary phase diagram (Figure 1A) shows that it enhances mixing of polar and non-polar components, although over a narrower composition range than ethanol.^9,13,14^ In contrast with water–ethanol–octanol systems, phase separation yields octanol-rich and (water + PAN)-rich phases (cf. Figure S1). Replacing the molecular co-solvent with an ionic liquid fundamentally changes phase behavior, leading to highly asymmetric partitioning between coexisting phases.

**Figure 1:**
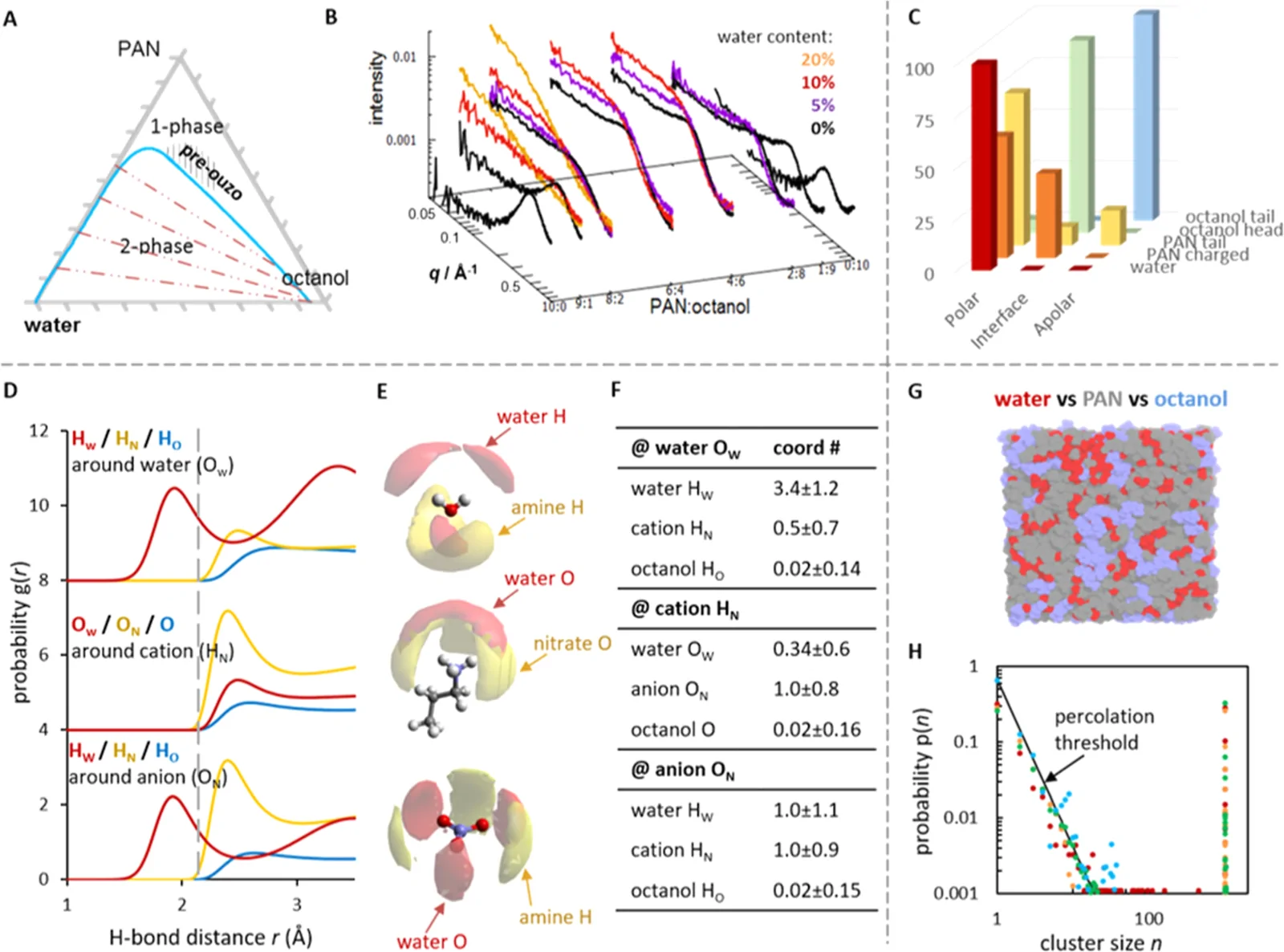
The water–PAN–octanol system: (A) Ternary phase diagram. Dotted lines show tie-lines between coexisting phases and shaded area shows the pre-ouzo region. (B) Small-angle X-ray scattering (SAXS) patterns in the single-phase region. (Figure S4 shows scattering patterns and compositions.) (C) Distribution of species across domains based on small-angle neutron scattering (SANS) invariant analysis of 20:60:20 wt % water/PAN:octanol. (D) atom–atom correlation functions, *g*_*ij*_(*r*), show H-bond distances. Gray dashed line distinguishes short from long hydrogen bonds. (E) 3D spatial distribution functions show 20% probability surfaces for H-bond acceptors and donors. (F) H-bond coordination numbers within 2.8 Å. (G) A snapshot of a converged simulation box with species color-coded. Rendered volumes approximate relative component abundance. (H) Cluster analysis of water (*O*_W_-*O*_W_, 0–3.4 Å), PAN head groups (N_O_–N_,_ 4.6 Å), PAN tails (C_M_-C_P_ or C_P_-C_P_, 4.7 Å), and octanol tails (C_8_–C_8_, C_X_-C_8_ or C_X_-C_X_, 4.7 Å) within the first coordination shell showing domains spanning simulation box. Solid line shows theoretical three-dimensional percolation threshold, p­(*n*) = *n* ^–2.2^.^50^

SAXS patterns (Figure 1B) show a non-monotonic evolution of low-q intensity consistent with the development of long-range but finite density fluctuations within a single phase commonly denoted as the “pre-ouzo” region in surfactant-free microemulsions. Critical compositions were not determined in this work. This describes structure at the nanometer scale, while neutron diffraction and SANS resolve molecular organization and compositional partitioning across domains. The amphiphilicity of PAN enables it to mix with long-chain alkanols by swelling its apolar domains and forming a periodic, bicontinuous nanostructure (indicated by the broad scattering peak)^41^ while preserving its polar H-bond network.^42^ Upon adding water, this gives way to an aperiodic structure indicative of fluctuation-dominated organization, also seen in other ultra-flexible microemulsions^14,43,44^ and some other IL-alcohol mixtures.^45–47^

SANS invariant analysis of chemically identical mixtures with multiple isotopic labeling^13^ reveals component distributions between domains. Figure 1C shows that the liquid structure can be described as consisting of a polar domain rich in water and PAN, an apolar domain of octanol chains, and an interfacial domain containing octanol hydroxyl groups and PAN. SANS results cannot be satisfactorily described by a two-domain invariant or by common structural models (see Supporting Information Section D, Figures S6–S8, and Tables S1–S4).

Neutron diffraction analyzed by empirical potential structure refinement^48^ resolves local molecular arrangements, showing that strong electrostatic and H-bonding interactions form a network within the polar domains. Water preferentially H-bonds to itself and nitrate (Figures 1D–F and S9 and S10), driving segregation of octanol into non-polar domains.^31,40,49^ The amphiphilicity of PAN stabilizes this nanostructure as its charged head groups are integrated into the polar network. Cluster analysis (Figure 1H and Supporting Information Section E) confirms the bicontinuous arrangement of polar components, with non-polar domains swollen by finite aggregates of octanol, also seen in a simulation snapshot (Figure 1G).

The amphiphilicity of PAN stabilizes nanoscale segregation in these mixtures, with the resulting distribution of species between domains presaging the onset of phase separation into an octanol-rich phase.

### Amphiphilic Ionic Liquid as a Polar Solvent

Replacing water instead with PAN as a polar solvent yields qualitatively different behavior. Mixtures of PAN with aliphatic alcohols can solubilize large amounts of hydrocarbons such as octane (Figures 2A, S3B). As in the water–PAN–octanol system, these mixtures show a periodic, microemulsion-like nanostructure that becomes fluctuation-dominated in the pre-ouzo region (Figures 2B, S5). Tie-lines in these systems, however, show a more equal distribution of octanol between coexisting phases than in water–PAN–octanol.

**Figure 2:**
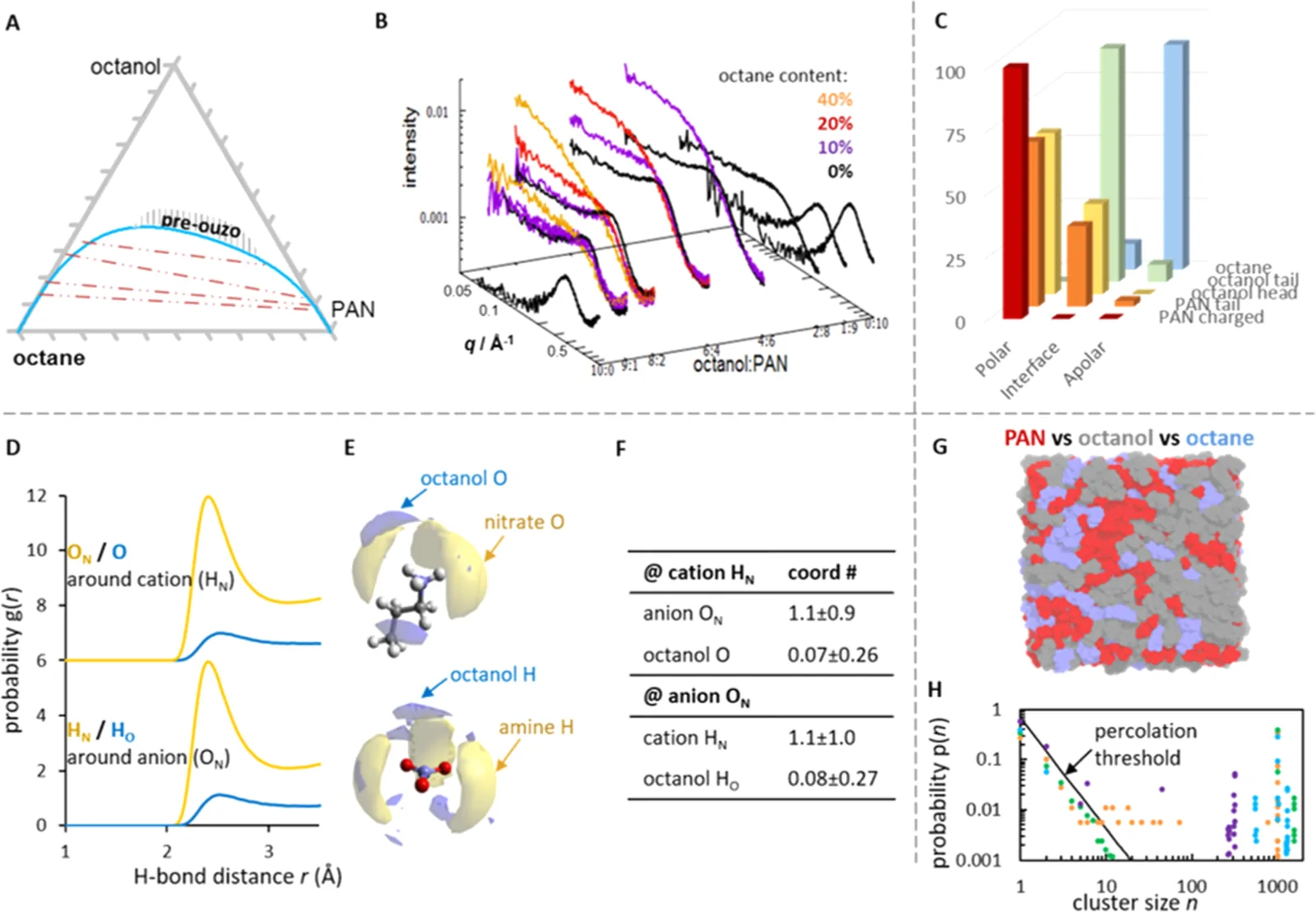
The PAN–octanol–octane system: (A) A ternary phase diagram. (B) SAXS patterns of single-phase samples. (C) Distribution of species across domains in 40:20:20 wt % PAN/octanol:octane. (D) Atom–atom correlation functions, *g*_*ij*_(*r*), showing H-bond distances. (E) 3D spatial distribution functions showing 20% probability surfaces for H-bond acceptors and donors. (F) Coordination numbers calculated within 0–2.8 Å. (G) A snapshot of a converged simulation box. (H) Cluster analysis of PAN head groups (N_O_–N_,_ 4.6 Å), PAN tails (C_M_-C_P_ or C_P_-C_P_, 4.7 Å), octanol tails (C_8_–C_8_, C_X_-C_8_, or C_X_-C_X_, 4.7 Å), and octane molecules (C_A_-C_A_, C_A_-C_T_, or C_T_-C_T_, 4.7 Å). Solid line shows theoretical three-dimensional percolation threshold, p­(*n*) = *n* ^–2.2^.^50^

Invariant analysis of multiple contrast SANS (Figure 2C) again shows the existence of three distinct domains. PAN, together with most of the octanol hydroxyl headgroups, forms a polar domain separated from octane in a distinct apolar domain. The majority of octanol tails form a continuous, octanol-rich interfacial region that stabilizes solubilization of the hydrocarbon.

Here, the polar network is stabilized by cation–anion electrostatic and H-bonding interactions (Figure 2D–F) similar to pure PAN and PAN–octanol mixtures.^31,42^ The amphiphilic nanostructure of PAN provides the scaffold for incorporation of octanol, which expands the PAN apolar domains.^42^ This enables the uptake of octane while preserving polar–apolar segregation. Cluster analysis and simulation snapshots (Figure 2G,H) show that both polar and apolar domains form continuous networks consistent with a bicontinuous, microemulsion-like structure.

This behavior shows that PAN can control the liquid nanostructure both as a hydrotrope and as an amphiphilic polar solvent capable of solubilizing non-polar oils. This extends surfactant-free microemulsions to fully non-aqueous systems and brings them closer to traditional microemulsions while removing the drawbacks of conventional surfactants.

### Non-amphiphilic Ionic Liquid as a Polar Solvent

The non-amphiphilic IL EtAN is immiscible with longer-chained alkanols than ethanol and lacks a polar–apolar domain structure. Hence, it can only replace water as the polar solvent in ternary mixtures with aliphatic alcohols, requiring either an ionic (e.g., PAN) or a molecular (e.g., ethanol) stabilizer. In EtAN–PAN–octanol mixtures (Figure 3A), the single-phase region is noticeably larger than its aqueous analogue (Figure 1A), but here too, phase separation leads to octanol-rich and polar, (EtAN + PAN)-rich phases. This partitioning is inconsistent with the approximately horizontal tie-lines observed in the water–ethanol–octanol system (Figure S1). The larger single-phase region reflects the greater compatibility of EtAN with PAN, allowing PAN to mediate mixing with octanol over a wider composition range than in the corresponding aqueous system.

**Figure 3:**
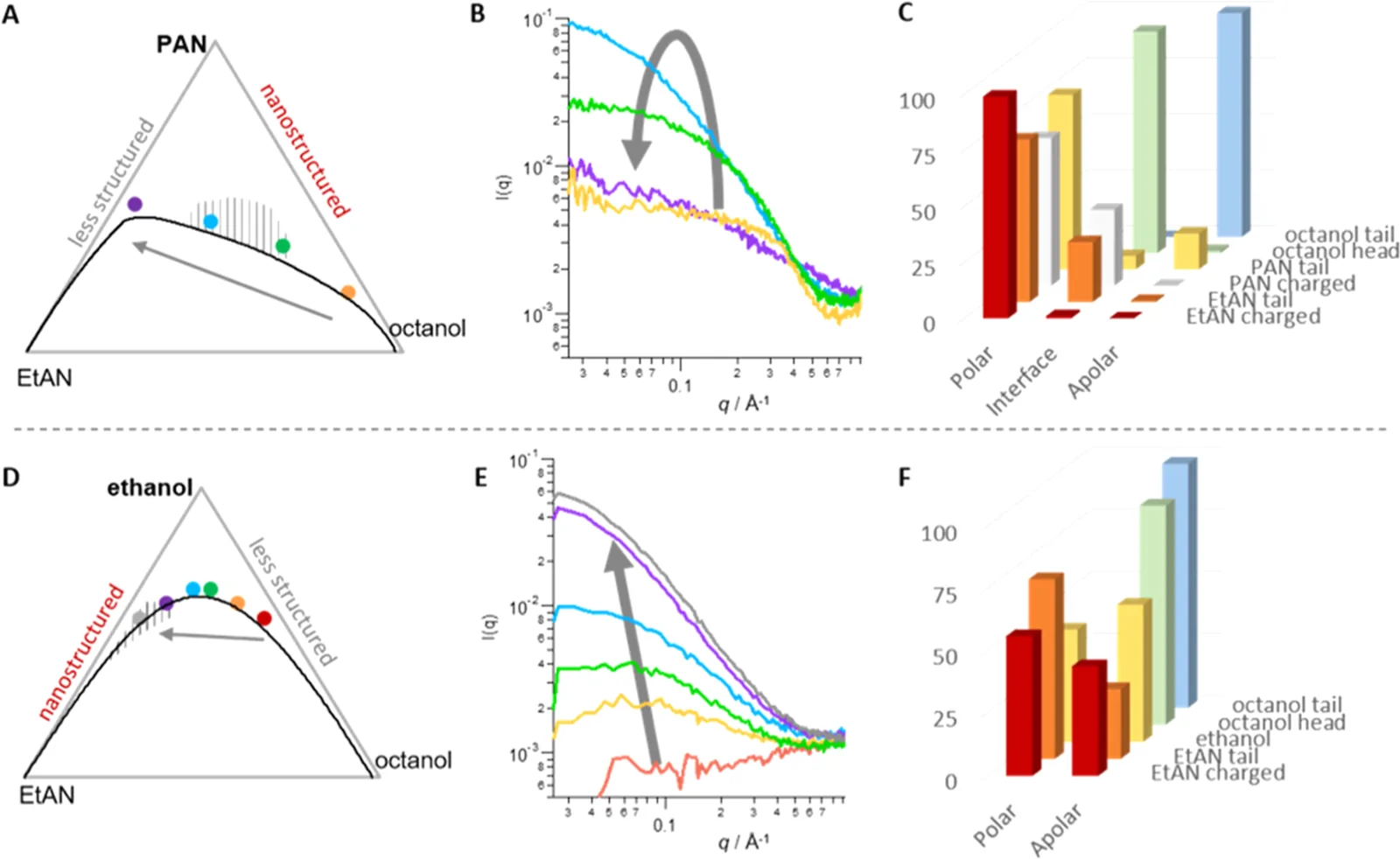
Ternary systems with EtAN as the polar component. (A&D) Ternary phase diagrams. Colored dots show compositions near the phase boundary, with corresponding SAXS patterns shown in (B&E). Distribution of species across domains in (C) EtAN/PAN:octanol 30:52.5:17.5 wt % and (F) EtAN/ethanol:octanol 30:60:10 wt %.

These mixtures lack the periodic liquid nanostructure except in the most PAN-rich samples far from the pre-ouzo region (Figure 3B). SANS analysis shows that, like water–PAN–octanol, the liquid nanostructure consists of octanol-rich and (EtAN + PAN)-rich domains separated by an interface enriched with the octanol hydroxyl group. EtAN functions as a non-amphiphilic polar solvent with a strong H-bond network, while amphiphilic PAN again stabilizes the liquid domain structure.

This distinction is further emphasized in EtAN–ethanol–octanol mixtures (Figure 3D,E). Here, the single-phase region is much smaller than in water–ethanol–octanol mixtures,^9,13,14^ no periodic nanostructure is evident, and the phase behavior differs markedly. The pre-ouzo region lies near the EtAN–ethanol axis, so phase separation leads to polar, EtAN-rich, and alcohol-rich phases. Without PAN, the liquid nanostructure can be described by only two domains (Figure 3F) consistent with the absence of an amphiphilic stabilizing interfacial region.

EtAN instead behaves as a conventional polar medium, highlighting the role of amphiphilicity in stabilizing the nanostructure and determining the coexisting phase compositions.

## Discussion and Conclusions

The amphiphilicity of the polar component controls the nanostructure and phase behavior in surfactant-free microemulsions. Amphiphilic polar components such as PAN act simultaneously as a polar solvent and structure-directing amphiphiles, generating a three-domain nanostructure with stabilized interfacial regions. In contrast, non-amphiphilic polar components such as water and EtAN yield a two-domain nanostructure and require a separate amphiphile to mediate mixing. This identifies amphiphilicity, rather than polarity, hydrogen-bonding capacity, or ionic character as the molecular parameter governing structure and phase stability.

Amphiphilicity enables ionic liquids such as PAN to stabilize surfactant-free microemulsions in mixtures of components with widely different polarities. This is a consequence of the pronounced nanostructure in the pure IL,^31^ by which it can direct molecular organization and distribution of other components including EtAN, water,^51^ alkanols,^41,42,52^ and alkanes while stabilizing the interfacial region. In contrast, a non-amphiphilic IL, EtAN, acts as a polar solvent with a dense electrostatic and H-bond network. Like water, it drives solvophobic segregation but cannot induce interfacial structure.

The role of octanol is distinct from that of PAN. Previous studies showed that octanol self-assembly in PAN is enabled by the underlying amphiphilic nanostructure of the ionic liquid, which stabilizes exposed octyl chains while preserving the polar ionic network.^42^ Here, octanol contributes directly to the interfacial region and facilitates hydrocarbon solubilization, but the emergence of the three-domain nanostructure depends on the amphiphilicity of PAN. Replacing PAN with the non-amphiphilic ionic liquid EtAN removes this structural motif despite the continued presence of octanol.

The behavior of these systems is thus similar to ultra-flexible microemulsions, in which such weakly amphiphilic compounds favor mixing by stabilizing a diffuse interfacial region. ILs extend this concept by embedding amphiphilicity within the polar solvent. This enables interfacial stabilization to emerge from their intrinsic polar–apolar domain structure, more closely resembling the role of surfactants in classical microemulsions. Although exemplified here using protic ionic liquids, this behavior is expected to apply generally to systems in which amphiphilicity is incorporated within the polar component.

Structure and stability can be tuned by changing the cation structure and composition. Increasing the proportion of purely polar components (water or EtAN) promotes expulsion of non-polar species, leading to phase separation. Increasing the fraction of amphiphilic IL stabilizes the nanostructure within the single phase. Similarly, changing the alcohol structure and content affects its partitioning and role in the interfacial region.

Changing the IL cation and anion structure provides additional means of control over the liquid nanostructure and stability.^21,22,53^ Longer cation chains enhance polar–apolar segregation and increase the characteristic length scale of the nanostructure, leading to greater solubilization capacity. Shorter alkyl chains reduce amphiphilicity,^34,54^ favoring homogeneous polar networks so that phase behavior approaches that of conventional polar–apolar mixtures.

The role of amphiphilic ILs has direct parallels with classical microemulsions, where surfactant structure and composition determine interfacial area and curvature, whence polar–apolar domain structure and solubilization capacity. Here, however, in contrast to conventional microemulsions, an interfacial structure is not imposed by a surfactant but emerges from the ionic liquid itself.

The strong coupling between domain structure and phase behavior has practical implications. The asymmetric partitioning of polar and non-polar components within the liquid suggests that such systems may be useful for liquid–liquid separations through control over phase equilibria.

Protic ionic liquids can combine the roles of polar solvent and surfactant as means to control nanostructure, solubilization, and coexisting phase compositions. More generally, amphiphilicity embedded within the solvent provides a direct route to designing nanostructured liquids with predictable structure and behavior.

## Supplementary Material



## Notes

Experimental details, phase diagrams, SAXS patterns, SANS invariant analysis, neutron diffraction data, EPSR refinements, cluster analyses, and supporting references (PDF)

## ⊥

ACS International Ltd, Binsey House, Wallbrook Court, North Hinksey Lane, Oxford OX2 0QS, UK
