# Supplementary material for: Amphiphilicity Controls Nanostructure in Surfactant-Free Ionic Liquid Microemulsions
Source: J Am Chem Soc. 2026 Sep 7;148(43):46802–8. doi: 10.1021/jacs.6c13294 (PMC13741835; doi:10.1021/jacs.6c13294)
Supplement: Supplementary file 1 [file ja6c13294_si_001.pdf]

Supporting Information for

Amphiphilicity Controls Nanostructure in Surfactant-Free Ionic Liquid Microemulsions

Haihui Joy Jiang,[a] Silvia Imberti,[b] Kathleen Wood,[c] Rob Atkin,[d] Gregory G. Warr*[a]

a) School of Chemistry and Sydney Nano Institute, The University of Sydney, NSW 2006, Australia

b) STFC, Rutherford Appleton Laboratory, Didcot, Oxfordshire OX11 OQX, UK

c) Australian Centre for Neutron Scattering, Australian Nuclear Science and Technology Organization, New Illawarra Road, Lucas Heights, NSW 2234, Australia

d) School of Molecular Sciences, The University of Western Australia, WA 6009, Australia

*E-mail: gregory.warr@sydney.edu.au

Table of Contents

A. Materials and Methods 3

B. Phase diagrams and miscibility comparisons 4

C. Scattering Patterns and Sample Compositions 6

D. SANS Invariant Analysis and Structural Models 10

E. Neutron Diffraction, EPSR Simulation, and Cluster Analysis 19

A. Materials and Methods

1-octanol (Ajax) and *n*-octane (Fluka) were obtained and used as received. Milli-Q water was used. Ethanolammonium nitrate and propylammonium nitrate were prepared based on previously published procedures.[1] Ethanolammonium nitrate (EtAN) and propylammonium nitrate (PAN) were synthesised by slow titration of the relevant amine base (propylamine from Acros, ethanolamine from Merck) into nitric acid (Ajax Chemicals). The reaction was maintained below 10 °C in an ice bath, and solution pH maintained between 5 and 6, preventing the formation of amine-related impurities. Products were dried first to below 5 wt% water content on a rotary evaporator, followed by drying under N_2 at 120 °C over 12 hours. Water contents were less than 0.2 wt% (typically much lower) by Karl Fischer titration after drying. EtAN melts at 52 °C but forms a long-lived supercooled liquid at room temperature, and that its melting point is substantially lowered in the presence of small amounts of water and other polar solvents.

Fresh deuterium oxide D_2O (Sigma, 99%) was added to a hydrogenous IL in a 10:1 molar ratio. The D_2O-IL mixture was dried on a rotary evaporator, followed by high-vac. This exchange was performed four times for each IL. ^{1}H-NMR revealed that, on average, 95% exchangeable hydrogens were replaced with deuterium. Non-exchangeable deuterated materials were purchased from Cambridge Isotopes.

Binary mixtures were prepared by mass in 2 mL glass vials at 5 wt% intervals. Samples were mixed by mechanical agitation on a Vortex mixer for three minutes, followed by bath sonication for 30 s. Miscibility was determined after 30 minutes equilibration and confirmed after at least one week. Selected samples were retained and remained unchanged over many months. Immiscible samples were identified when observing phase separation, or optical turbidity when mechanically agitated. Phase boundaries of ternary mixtures were determined by titration of a third component into the binary mixture at 2 wt% intervals until cloudiness appeared. Selected ternary mixtures within the two-phase region were prepared, mixed, and left to separate into two liquid layers for at least 72 h. The composition of each layer was analysed by ^{1}H-NMR 300MHz integration. All measurements were done at 25 ºC. Selected samples prepared by different protocols or retained for several weeks showed the same scattering patterns and phase behaviour.

Small- and Wide-Angle X-ray Scattering (SWAXS) was performed on an Anton Paar SAXSess with point collimation of a rotating anode CuK_α (1.541Å) source. Samples were measured at 25 °C in 1mm quartz capillaries for five minutes. A single image plate was used and radially averaged normalised to the direct beam. Empty cell scattering was subtracted, and all patterns were calibrated to water.

SANS was performed at the QUOKKA beamline[2] at Centre for Neutron Scattering (ANSTO, Australia) using 1mm path-length cylindrical Hellma Cells. A neutron wavelength of 5 Å was used with sample-to-detector distances of 1.3m and 8m, providing a q range of 0.01 to 0.7 $Å^{-1}$. Raw SANS data were reduced to 1D in IGOR Pro with reduction procedure provided by NIST modified for uses on QUOKKA.[3]

Neutron liquid diffraction was measured on the SANDALS beamline at the Rutherford Appleton Laboratory (UK). SANDALS is a time-of-flight diffractometer that measures the static structure factor, S(q), over a q range of 0.2 - 50 $Å^{-1}$. Samples were loaded into 1mm path length, 35 x 35 mm^2 $Ti_{0.68}Zr_{0.32}$ sample cells, which is both chemically inert and null scattering. Measurements were performed under vacuum at 25 °C, and converted to absolute units via empty cell, empty instrument and a vanadium standard using the GUDRUN program[4]. Samples were weighed before and after experiment to ensure no

loss had occurred. Diffraction patterns from chemically identical but multiple different isotopic (H/D) labels was fit using Empirical Potential Structure Refinement (EPSR) developed for molecular liquids and disordered systems.[5-8]

B. Phase diagrams and miscibility comparisons

This section corresponds to the manuscript references comparing the water-PAN-octanol system to conventional hydrotropic systems (e.g., water-ethanol-octanol), as well as variations involving other alkanols and EtAN.

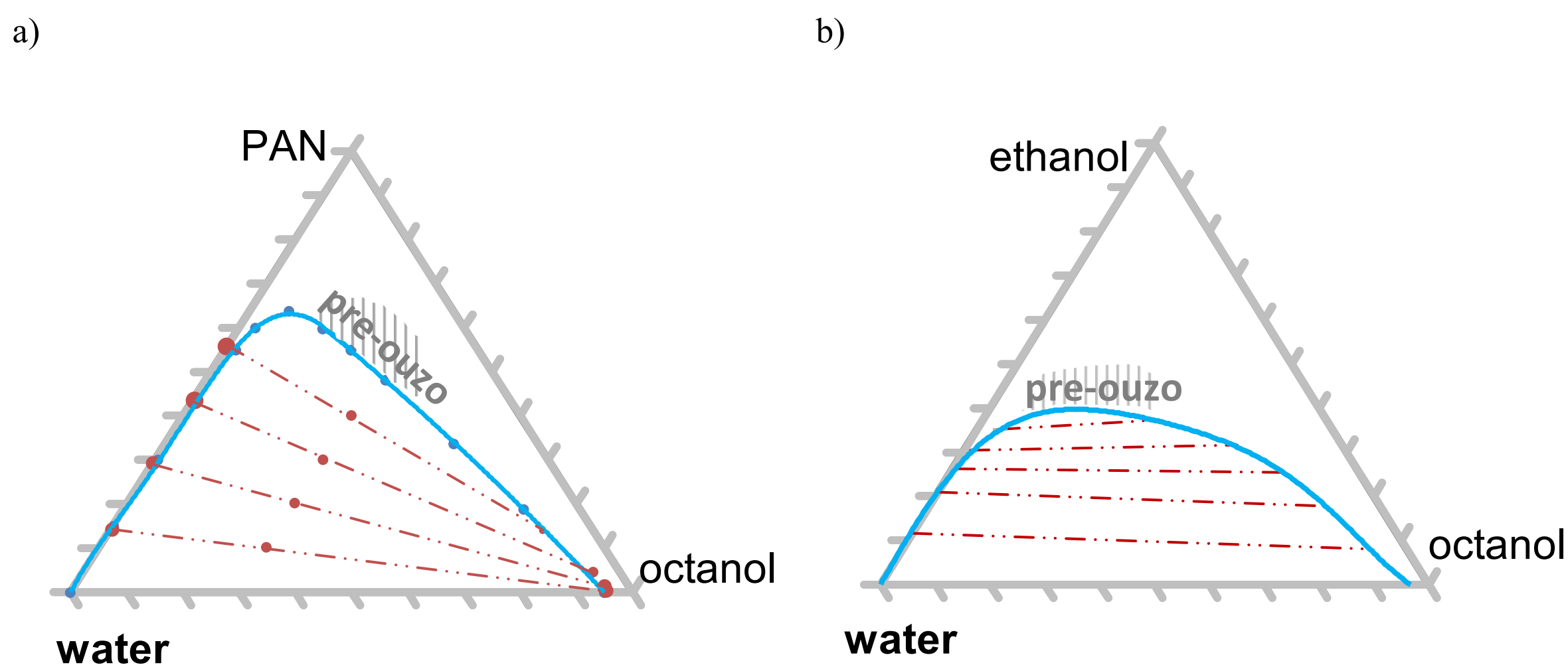

Figure S1. Ternary phase diagrams of a) water-PAN-octanol and b) water-ethanol-octanol at 298K.[9] Solid lines represent the phase boundary, dotted lines represent tie-lines in the two-phase region.

The phase behavior of the water-PAN-octanol system is similar to conventional hydrotropic systems, such as the water-ethanol-octanol system, shown in Figure S1.[10] Hydrotropes, such as ethanol, are industrial solubilisers for organic solutes in water.[11] Compared to surfactants, they are weakly amphiphilic with a smaller hydrophobic region. As a result, solubilisation of non-polar solutes normally requires high hydrotrope levels.[12] PAN is comparable to ethanol as both are small amphiphiles. The difference is that water-PAN-octanol has a wider two-phase region and its phase boundary is more asymmetric than water-ethanol-octanol. Tie-lines in water-PAN-octanol are highly tiled, whereas tie-lines in water-ethanol-octanol are relatively flat. When using ethanol as the amphiphile, there is a near-equal distribution of ethanol in water and octanol.

Figure S2 shows four systems with either water or EtAN as the polar component, and with either ethanol or PAN as the amphiphile. The phase boundary shifts depending on the combination. Shown in a), if starting from ethanol-octanol molecular mixtures, the system can accommodate more water than EtAN. Similar to a salting-out effect, the addition of a non-amphiphilic ionic liquid decreases miscibility. Shown in b), if starting from PAN-octanol mixtures, which is an ionic environment, the system can better accommodate an additional ionic liquid, EtAN, than water. Furthermore, examining the tie-lines reveals distinct extraction behaviors: in the EtAN-ethanol-octanol system, the tie-lines in the two-phase region are highly tilted with

positive gradients, meaning an immiscible sample phase separates into an EtAN-rich layer and an ethanol/octanol-rich layer. Conversely, in the EtAN-PAN-octanol system, the tie-lines exhibit negative gradients, leading to separation into an EtAN/PAN-rich phase and a purely octanol-rich phase.

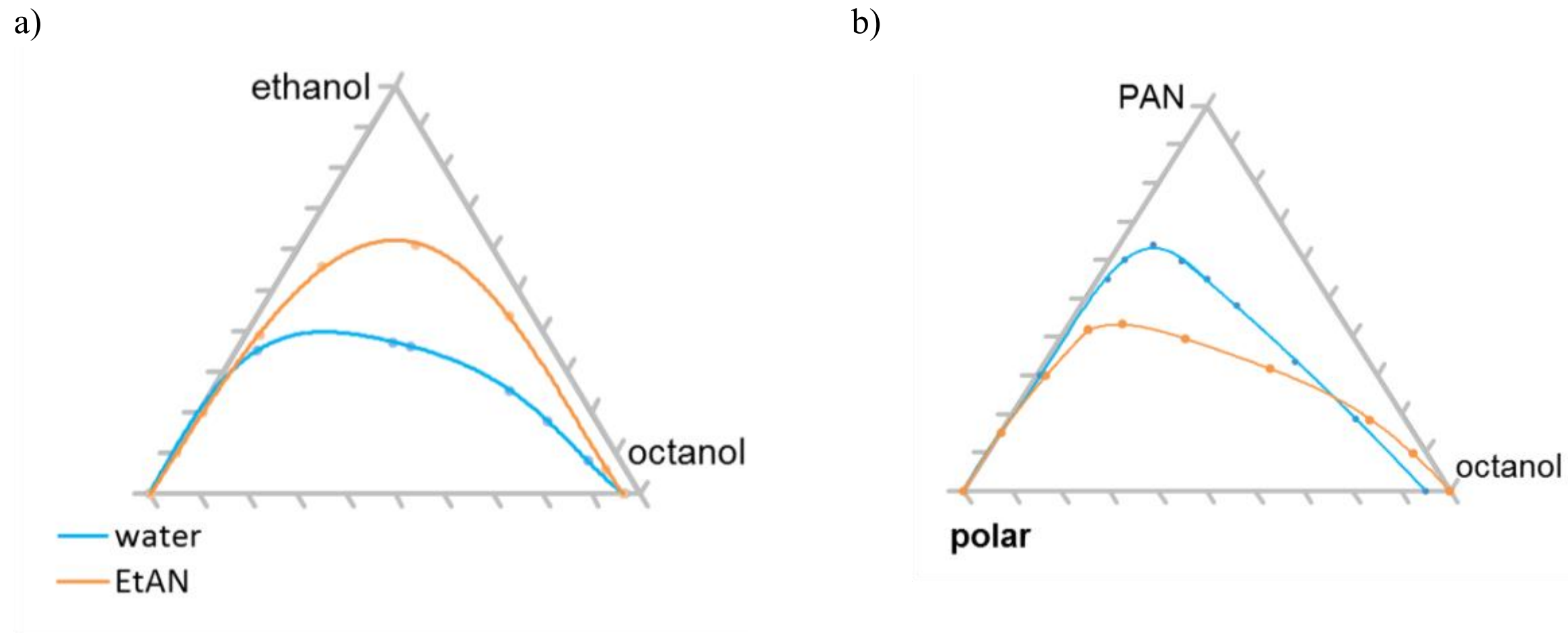

Figure S2. Ternary phase diagrams (wt%) of polar–n-octanol–amphiphile systems at 298K. A comparison between water and EtAN ternary systems. a) using ethanol as the amphiphile, b) using PAN as the amphiphile. — water as the polar component; — EtAN as the polar component.

By varying the alcohol chain length, as shown in Figure S3b, results demonstrate the influence of alcohol amphiphilicity on its ability as a solubiliser. Short chain alcohols such as butanol (C4OH) are better at solubilising IL than oil, therefore the phase boundary of PAN-butanol-octane is left-skewed. As the alkyl chain length increases, the alcohol solvophobicity increases and becomes better at solubilising oil than IL, therefore the phase boundary of PAN-decanol-octane is right-skewed.

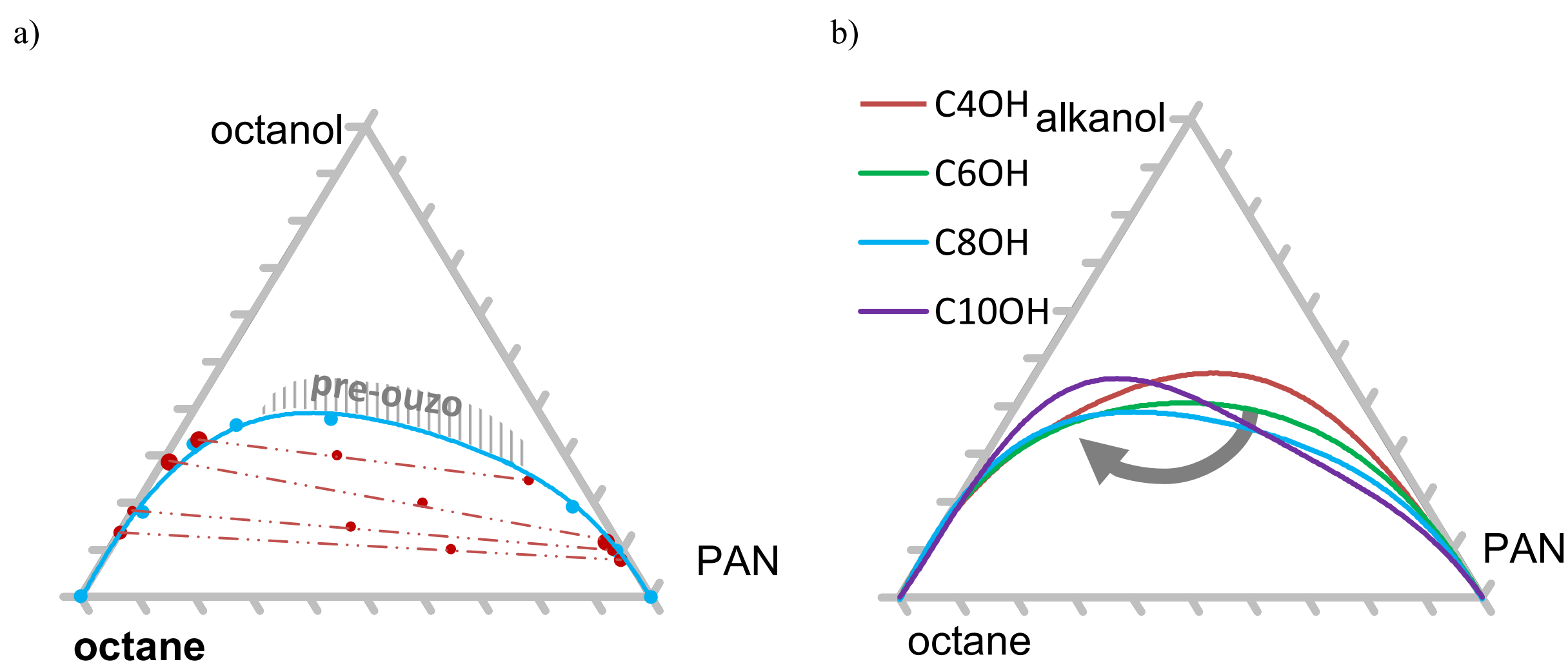

Figure S3. Ternary phase diagrams (wt%) of octane-alkanol-PAN systems at 298K. a) octane-octanol-PAN ternary system with tie-lines. b) octane-alkanol-PAN systems, as a function of alkyl chain ratio. Experimental errors are represented by the radius of data points.

C. Scattering Patterns and Sample Compositions

This section contains the detailed SWAXS profiles for the ternary mixtures, demonstrating non-monotonic structural changes.

Figure S4a is the scattering pattern of PAN-octanol mixtures without the addition of water.[13] The presence of low-angle scattering means these binary solutions contain aggregates at the nanoscale. Scattering intensity, indicating the extent of the structure, changes non-monotonically as going from PAN-rich to octanol-rich samples. Peak position ranges between 0.24-0.51 Å^{-1}, suggesting a correlation length of 12-26 Å based on Brag's law of $d=2\pi/q$. The nanostructure detected from PAN-octanol is larger than pure PAN, which has a sponge-like structure with repeating spacing of 11.6Å, approximately the length of two propyl chains.[14] Experimental evidences show that liquid structure is sensitive to formulation. The most intense low-q scattering occurs precisely in the 'pre-ouzo' region (single-phase compositions approaching the binodal that exhibit enhanced but finite composition fluctuations), indicating that the addition of the polar solvent modifies and enhances the underlying ionic liquid structures without abrupt changes to structural periodicity.

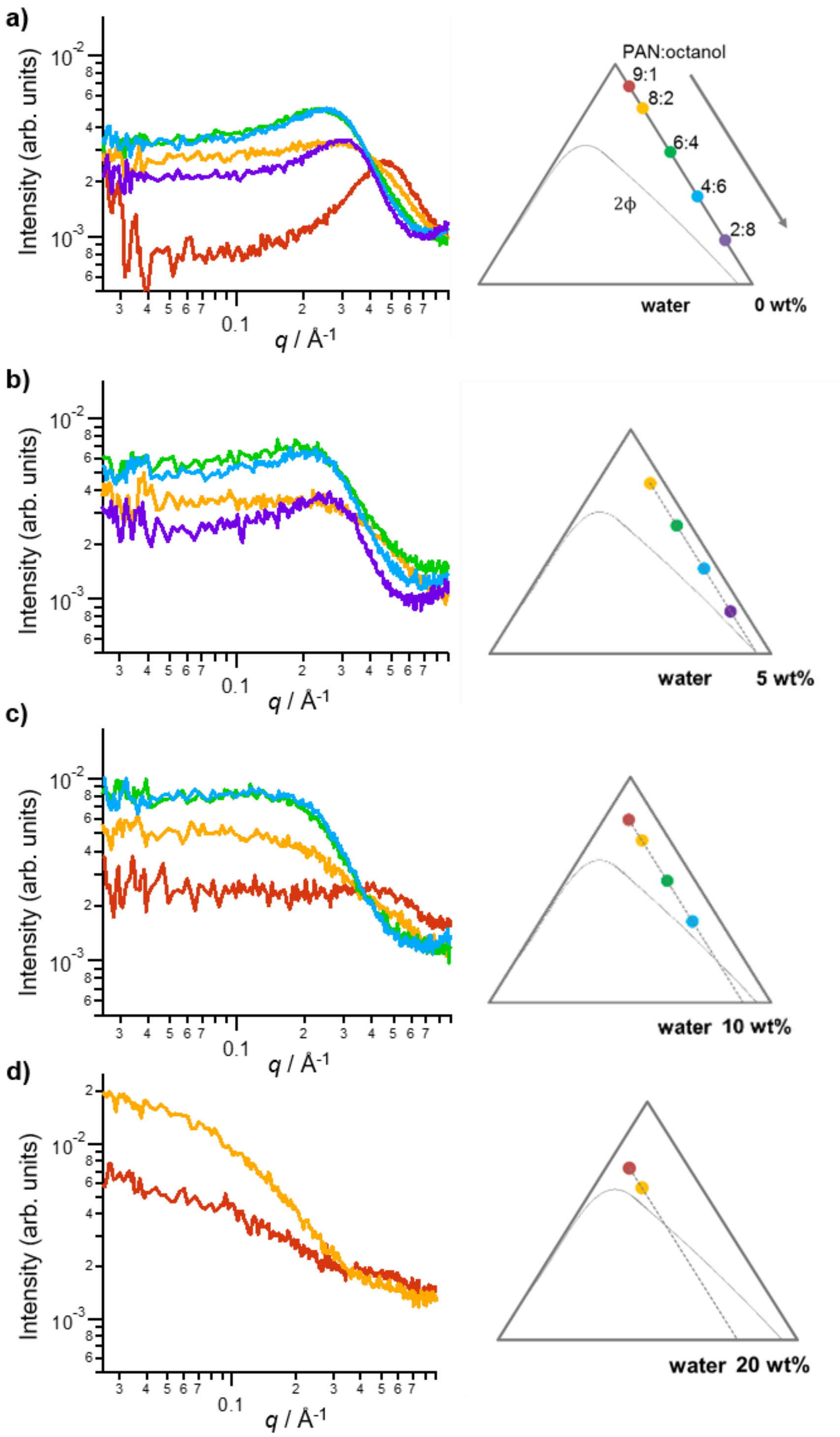

Figure S4. SAXS of water–n-octanol–PAN mixtures at various compositions at 298K. Water content increases from 0 (ref 17), 5, 10 to 20wt% (top to bottom); At any given water content, PAN:octanol ratio varies as labelled on its corresponding phase diagram.

Unlike the aqueous systems where water is accommodated into PAN-rich regions, Figure S5 shows that octane is accommodated into the octanol-rich compositions. The structure of these ternary PAN-octanol-octane mixtures (Figure S5b-d) remains closely related to the underlying PAN-octanol binary formulation (Figure S5a). As the octane concentration increases, there is a pronounced increase in low-q scattering intensity, which signifies a substantial enhancement in the nanoscale structure. These scattering profiles demonstrate that PAN effectively supports the nanoscopic segregation of octanol and octane—a capability lacking in purely molecular equivalent mixtures (such as ethanol-octanol-octane). The strong amphiphilicity of PAN drives the arrangement of the alcohol at the interface, which in turn enables the robust solubilization of the non-polar oil.

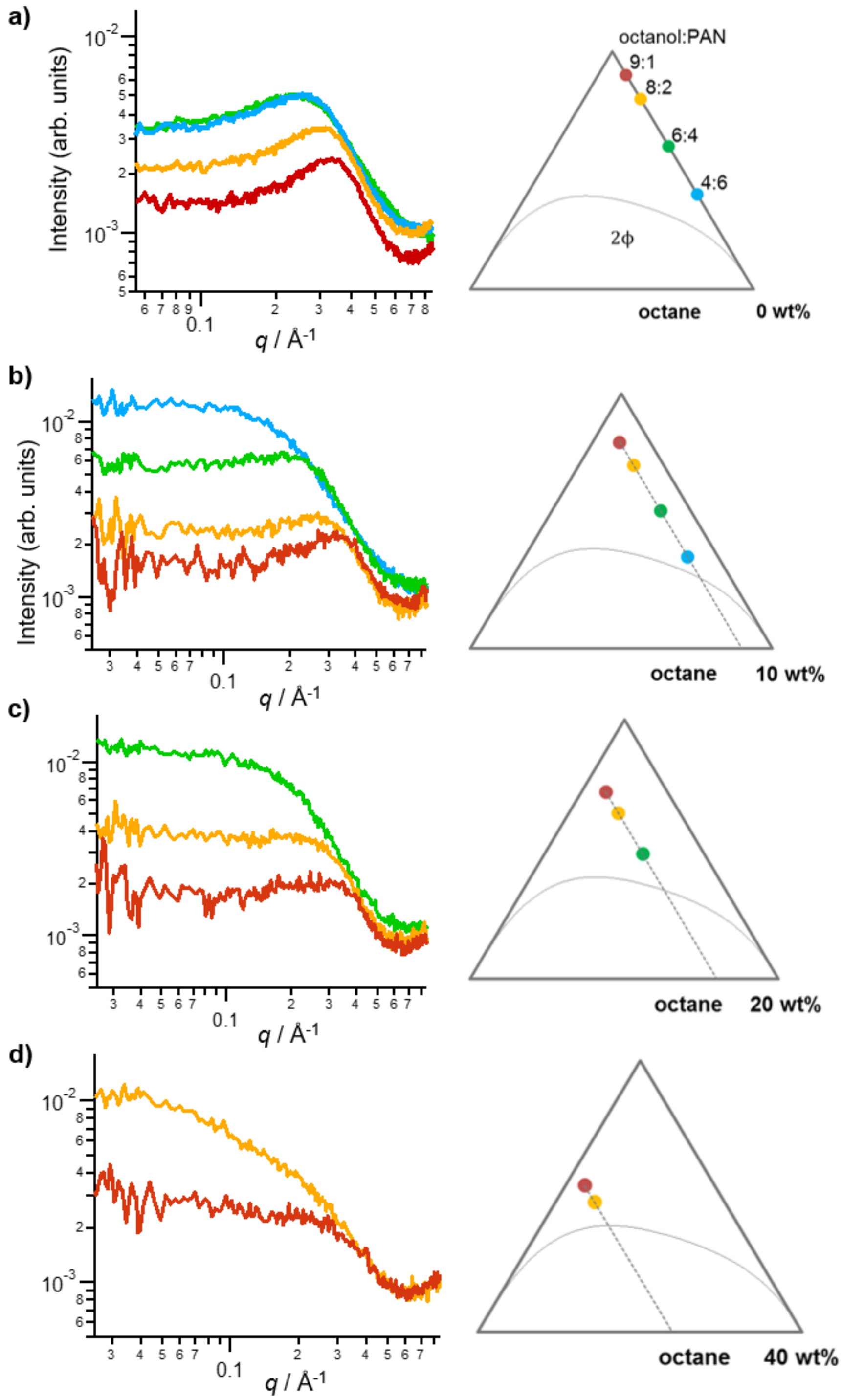

Figure S5. SAXS of octane–n-octanol–PAN mixtures at various compositions at 298K. Octane content increases from 0, 10, 20 to 40wt% (left to right); At any given octane content, PAN:octanol ratio varies as labelled on its corresponding phase diagram.

D. SANS Invariant Analysis and Structural Models

In contrast to X-ray scattering, where radiation interacts with electrons, neutrons interact with nuclei of atoms. Therefore, they can distinguish between different isotopes, most notably hydrogen and deuterium. This allows the study of complex multiphase systems by isotopic substitution. In this project, small angle neutron scattering (SANS) experiments are designed with a q range from 0.7 $Å^{-1}$ to 0.01 $Å^{-1}$, corresponding to 9 Å to 600 Å in real space.

For neutrons, the scattering length density (SLD), is defined as:

$$SLD = \frac{\sum_{i=1}^{n} b_i}{v_m} \qquad [1]$$

$$v_m = \frac{N_A \cdot \rho}{M_W} \qquad [2]$$

where b_i is the bound coherent scattering length of the atom_i in a molecule: and v_m is the molecular volume, which is calculated based on density ρ, molecular weight M_W, and the Avogadro constant N_A.

To further derive the SLD of the individual functional group, we refer to Edward's Equation:[15]

$$V_M = 16.63n + 32.57 \qquad [3]$$

where V_M (mL/mol) is the molar volume for an alkyl tail, and n is the number of methylene units and the constant (32.57) accounts for the volume of the terminal methyl group.

Edward's equation approximates the volume of a hydrocarbon tail in liquid mixtures. Assuming there is no volume change during mixing, the partial volume of the individual functional group is calculated accordingly. Note that results from Edward's equation are comparable to Gruen's volume of chains in amphiphilic aggregates, with negligible differences (<1%) at RT.[16] The densities of hydrogenous compounds were experimentally measured by a densitometer.

SLDs for all components used in this study are shown in Table S1. Values were calculated using the National Institute of Standards and Technology (NIST) SLD calculator.

Table S1. SLD values from NIST SLD calculator[17] for all components used in this study. These values are used for subsequent invariant analysis.

Component	Chemical Formula	Density (g/cm^3)	SLD (10^{-6} Å^{-2})
Water	H_2O	1.00	-0.56
heavy water	D_2O	1.11	6.36
Ethanol	C_2H_6O	0.79	-0.35
D6-ethanol	C_2D_6O	0.89	6.10
ethanol head	OH	1.85	1.35
D1-ethanol head	OD	1.96	8.18
ethanol tail	C_2H_5	0.59	-0.66
D5-ethanol tail	C_2D_5	0.69	5.71
EtAN	$C_2H_8N_2O_4$	1.38	1.70
D4-EtAN	$C_2H_4D_4N_2O_4$	1.42	4.48
EtAN charged	NH_3NO_3	1.67	3.16
D3-EtAN charged	ND_3NO_3	1.73	7.12
EtAN tail	C_2H_5O	1.05	0.06
D1-EtAN tail	C_2H_4OD	1.09	1.53
PAN	$C_3H_{10}N_2O_3$	1.16	1.01
D3-PAN	$C_3H_7D_3N_2O_3$	1.19	2.86
PAN charged	NH_3NO_3	2.00	3.80
D3-PAN charged	ND_3NO_3	2.08	8.57
PAN tail	C_3H_7	0.66	-0.57
Octanol	$C_8H_{18}O$	0.82	-0.32
D1-octanol	$C_8H_{17}OD$	0.83	0.08
D17-octanol	$C_8D_{17}OH$	0.93	6.43
octanol head	OH	1.86	1.36
D1-octanol head	OD	1.99	8.30
octanol tail	C_8H_{17}	0.76	-0.42
D17-octanol tail	C_8D_{17}	0.87	6.73
octane	C_8H_{18}	0.70	-0.52
D18-octane	C_8D_{18}	0.81	6.42

Initial attempts to fit the SANS data using standard structural models, such as the Debye-Anderson-Brumberger (DAB) model, polydisperse spheres, and bi-continuous structures, were unsatisfactory for the pre-ouzo region because these surfactant-free mixtures lack a classical compact surfactant monolayer. For example, fitting the water-PAN-octanol system (20 wt% water in octanol–PAN) with the DAB model yielded unacceptably large χ^2 values for several contrasts (Table S2), demonstrating that a single geometric model could not systematically fit all contrasts and compositions.

Table S2. DAB model fitting of water-PAN-octanol systems.

20wt% water in octanol–PAN(1:3 by wt)			
Contrast	H_2O–$C_8\mathbf{D_{17}}OH$–$C_3H_7NH_3NO_3$	$\mathbf{D_2}O$–$C_8\mathbf{D_{17}}OH$–$C_3H_7N\mathbf{D_3}NO_3$	$\mathbf{D_2}O$–$C_8H_{17}O\mathbf{D}$–$C_3H_7N\mathbf{D_3}NO_3$
q-range (Å^{-1})	0.00914 - 0.34	0.00914 - 0.26	0.00914 - 0.35
Background	0.65	0.339	0.7
Scale	0.0206	0.0038	0.0073
Length (Å)	7.3	8.44	9.34
Chi^2	21.6	10.8	218

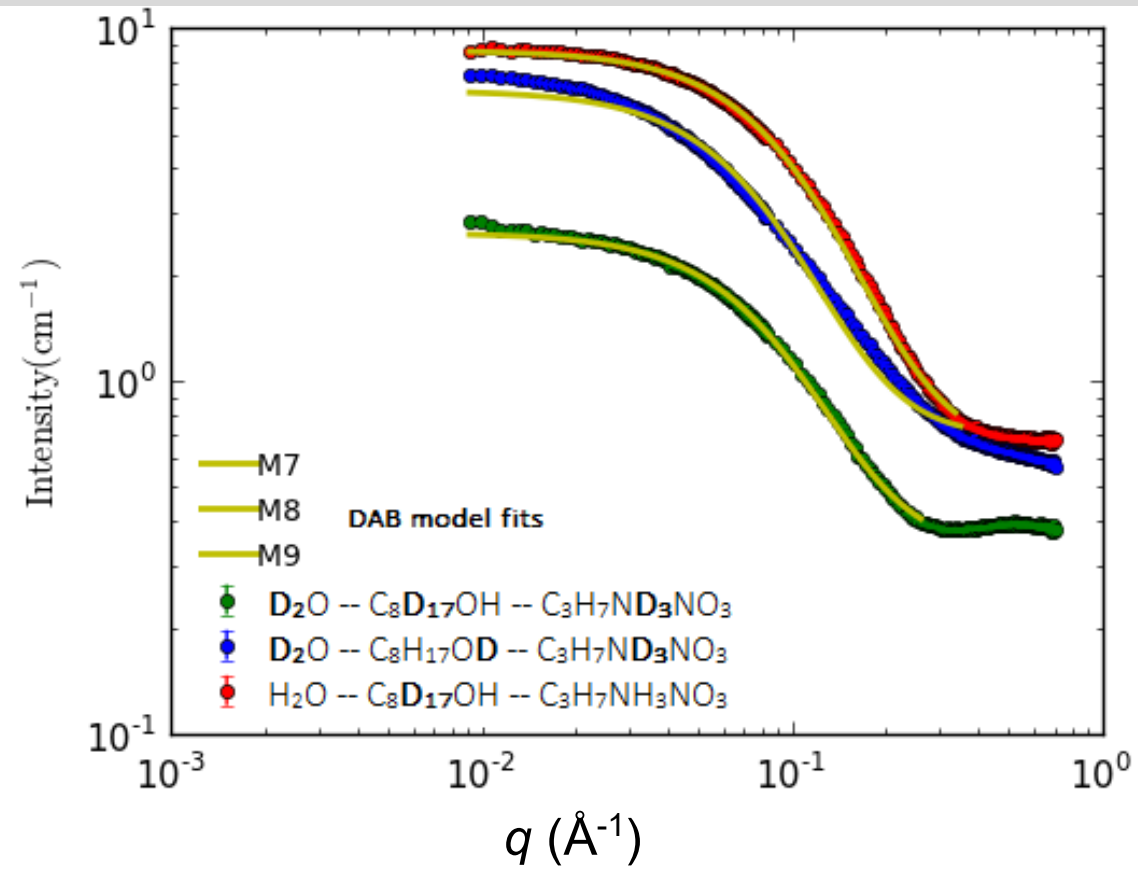

10wt% water in octanol–PAN(1:3 by wt)			
Contrast	H_2O–$C_8\mathbf{D_{17}}OH$–$C_3H_7NH_3NO_3$	$\mathbf{D_2}O$–$C_8\mathbf{D_{17}}OH$–$C_3H_7N\mathbf{D_3}NO_3$	$\mathbf{D_2}O$–$C_8H_{17}O\mathbf{D}$–$C_3H_7N\mathbf{D_3}NO_3$
q-range (Å^{-1})	0.00914 - 0.4	0.014 - 0.32	0.012 - 0.5
Background	0.434	0.23	0.567
Scale	0.05	0.0204	0.0545
Length (Å)	3.28	3.4	2.397
Chi^2	59	8	12.1

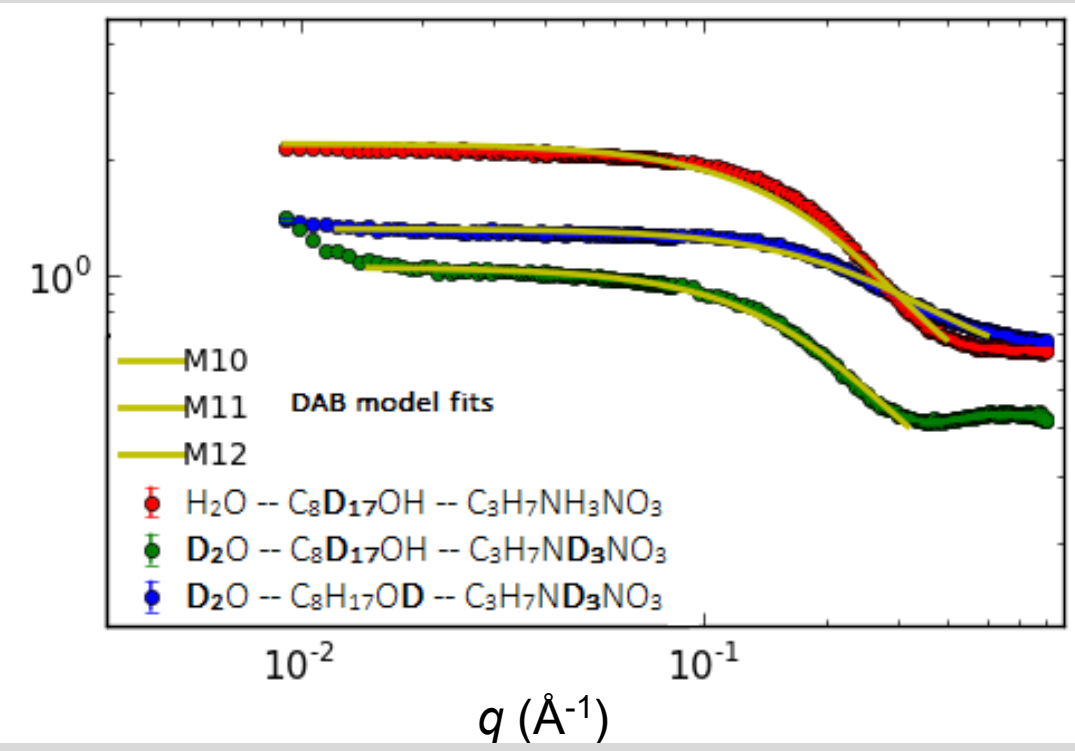

octanol–PAN(1:3 by wt)			
Contrast	$C_8\mathbf{D_{17}}OH$–$C_3H_7NH_3NO_3$	$C_8\mathbf{D_{17}}OH$–$C_3H_7N\mathbf{D_3}NO_3$	$C_8H_{17}O\mathbf{D}$–$C_3H_7N\mathbf{D_3}NO_3$
q-range (Å^{-1})	0.00914 - 0.45	0.014 - 0.35	0.00914 - 0.7012
Background	0.488	0.301	0.688
Scale	0.05	0.035	0.0689
Length (Å)	2.5	2.5	1.5
Chi^2	66	12.2	34.6

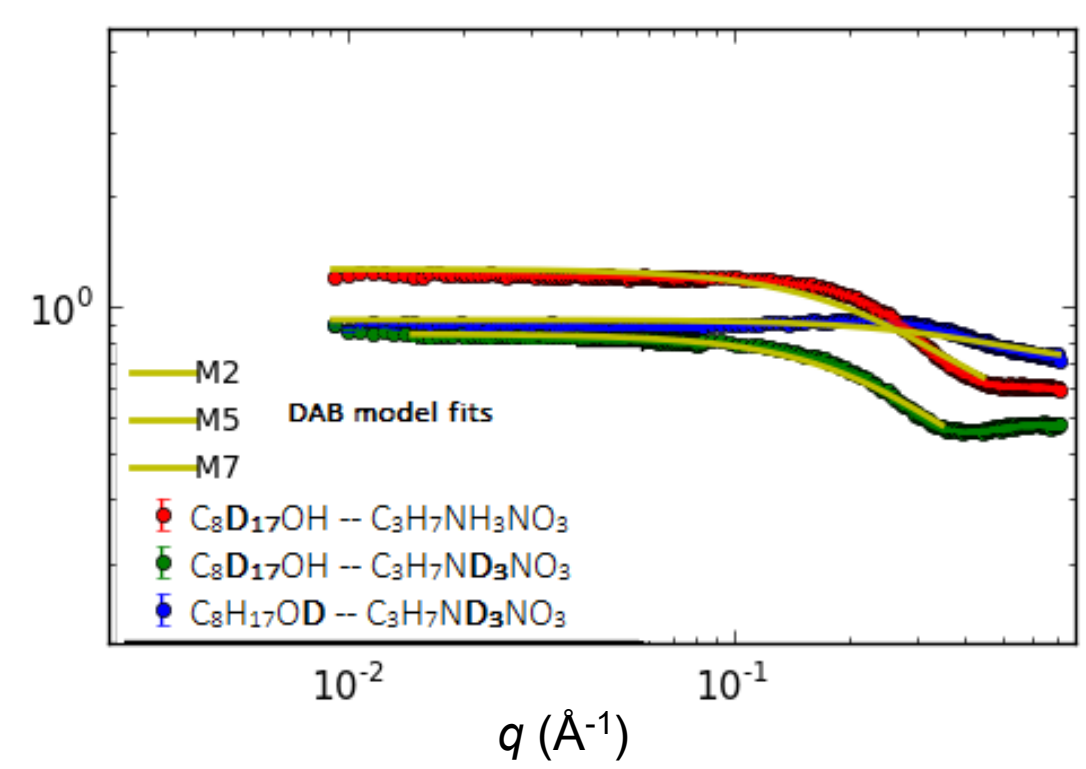

Furthermore, because the coherent and incoherent Scattering Length Densities (SLDs) differ significantly for each component (e.g., the coherent SLD of D_2O is much higher than d4-EtAN), the resulting contrast is not linearly related to the functional group distribution in Fourier space. Therefore, a structure-independent, 'model-free' invariant analysis was adopted to simultaneously fit multiple isotopic contrasts (Tables S3-4). This method allows the selective isolation of molecular components contributing to the nanostructure. We consider two immiscible components to form separate domains, and the amphiphilic molecule is either partitioning in between (i.e., a two-domain model) or accumulating at the interface and forming its own domain (i.e., a three-domain model). By minimizing the difference between experimental and theoretical invariants (Δ), we generate a distribution of individual components in each domain.

Table S3. Invariant analysis of "polar" ternary mixtures using two-domain and three-domain models.

a)	water	PAN charged	PAN tail	octanol head	octanol tail	% diff	HPAN-D17oct-H2O	D3PAN-D17oct-D2O	D3PAN-D1oct-D2O
2-domain	100	93	45	67	1	31	94	0	0
	0	7	55	33	99				
3-domain	100	59	74	7	0				
5-variable	0	41	9	94	0	16	48	0	0
	0	0	17	0	100				

b)	EtAN charged	EtAN tail	PAN charged	PAN tail	octanol head	octanol tail	% diff	HPAN-D17oct-HEtAN	D3PAN-D17oct-	D3PAN-D1oct-D4EtAN
3-variable	91	91	93	93	20	20	54	52.65	9	99
	9	9	7	7	80	80				
4-variable	100	100	100	47	0	0	43	96	0	32
	0	0	0	53	100	100				
3-domain	99	72.6	66	78	0	0				
5-variable	1	26.8	34	6	99	0	15	41	2.7	2.3
	0	1	0	16	1	100				

c)	EtAN charged	EtAN tail	ethanol head	ethanol tail	octanol head	octanol tail	% diff	D4EtAN-D6eth-D1oct	D4EtAN-Heth-D17oct	HEtAN-D6eth-D17oct
3-variable	3	3	70	40	32	32	9	22	5.2	0.1
	97	97	30	60	68	68				
4-variable	78	78	4	22	56	56	0	0.57	0.19	0.001
	22	22	97	78	44	44				
5-variable	56	72	45	45	15	2	0	0.003	0.02	0.009
	44	28	56	56	85	98				

Table S4. Invariant analysis of "apolar" ternary mixtures using two-domain and three-domain models.

	PAN charged	PAN tail	octanol head	octanol tail	octane	% diff	D3PAN-D1oct-Hoctane	HPAN-Hoct-D18octane	HPAN-D17oct-D18octane	HPAN-D17oct-Hoctane
2-domain	100	75	80	6	5	53	3.5	97	3	56
5-variable	0	25	20	94	95					
3-domain	1	1	11	11	100					
3-variable	99	99	31	31	0	27	82	7	0	0
	0	0	58	58	0					
3-domain	100	66	64	0	0					
5-variable	0	32	36	93	10	0	0	0	0	0
	0	2	0	7	90					

The theory behind the invariant analysis is that, in the system with clear interface between particles and surrounding solvent, Porod's law states the scattering intensity becomes proportional to q^{-4}, as q tending to infinity.[18]

Porod's law defines:

$$I(q) \approx \frac{2\pi}{q^4} \rho^2 S \quad , \quad (q \rightarrow \infty) \qquad [4]$$

where S is the particle surface; q is the scattering vector, and ρ is the scattering length density of the system.

Porod's invariant is an important overall value computed from the scattering experiment. The invariant depends only on the particle volume, not on its form.[19] The experimental invariant Q^*_{exp} is defined as:

$$Q^*_{\exp} = \int_0^\infty I(q) q^2 \, dq \qquad [5]$$

where I(q) is the scattering intensity after background subtraction, and q is the scattering vector.

The scattering background is calculated based on Porod's Law of $I(q) q^4 \propto q$. The integration range is approximated by plotting $I(q) q^2$ against q.

For an incompressible, two-domain system, the theoretical invariant Q^*_{theory} is defined as:

$$Q^*_{theory} = 2\pi^2 \Delta\rho^2 \varphi(1-\varphi) \qquad [6]$$

where φ is the volume fraction of $domain_A$, (1- φ) is the volume fraction of $domain_B$, and $\Delta\rho$ is the contrast difference calculated based on the total SLD of $domain_A$ (ρ_A) and $domain_B$ (ρ_B):

$$\Delta\rho = |\rho_A - \rho_B| \qquad [7]$$

This assumes each domain is homogeneous, and the domain SLD is calculated based on individual SLDs and corresponding volume fractions of its components. However, if one of the two domains is not homogeneous and contains aggregates of a specific species, the theoretical invariant calculated based on Equations [8] and [9] may not match with the experimental invariant. In this case, we consider a three-domain system[20]:

$$Q^*_{theory} = 2\pi^2 \times (\varphi_A(\rho_A - \bar{\rho})^2 + \varphi_B(\rho_B - \bar{\rho})^2 + \varphi_C(\rho_C - \bar{\rho})^2) \qquad [8]$$

where A, B, and C represent three types of domains within the sample, and $\bar{\rho}$ is the average SLD of the sample, calculated by:

$$\bar{\rho} = \varphi_A\rho_A + \varphi_B\rho_B + \varphi_C\rho_C \qquad [9]$$

Invariant analysis starts from a two-domain model with a small number of variables and rationally moves to a more complex model (three-domain) to better describe SANS data when necessary (Figure S6). In cases where experimental data shows strong evidence of an additional type of segregation that cannot be captured by the two-domain model, increasing the number of variables to utilize the three-domain model significantly reduces the average error. For instance, in the EtAN-PAN-octanol system, a 3-variable, 2-domain model resulted in an average Δ of 54%, whereas a 5-variable, 3-domain model reduced this to 15% (Figure S6c). There is a similar significant improvement in the agreement between experimental and theoretical invariants when going from a 2-domain model (polar–apolar) to a 3-domain model (polar–interface–apolar) for systems like water-PAN-octanol (Δ reduced from 31% to 16%; Figure S6b) and PAN-octanol-octane (Δ reduced from 53% to 0%).

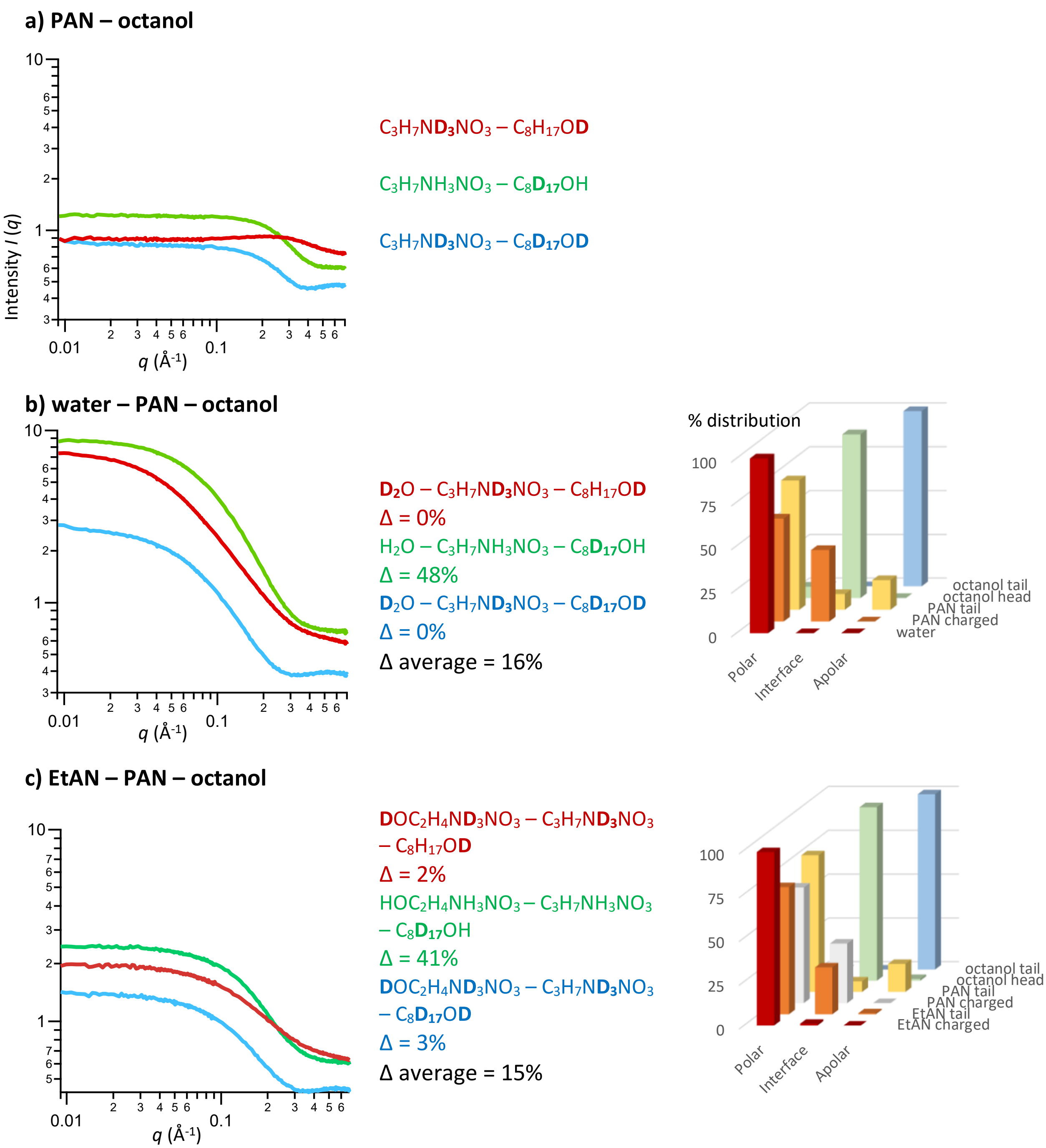

Figure S6 PAN–octanol with an additional polar component; SANS patterns with multiple contrasts at 298K (labels shown in colour), their corresponding Δ (% difference between experimental and theoretical invariant), and the distribution of species across domains. a) PAN–octanol (3:1), b) water–PAN–octanol (20:60:20), and c) EtAN–PAN–octanol (30:52.5:17.5). Ratios by wt. based on the fully hydrogenous sample. Deuterated samples have the same molar composition.

However, for the EtAN-ethanol-octanol system (Figure S7b), fitting the data with a three-domain model did not provide additional information, as the initial two-domain model already yielded an average Δ of

0%, indicating the 'apolar' domain may be structurally inhomogeneous with non-negligible amounts of EtAN charged groups present alongside the apolar hydrocarbons.

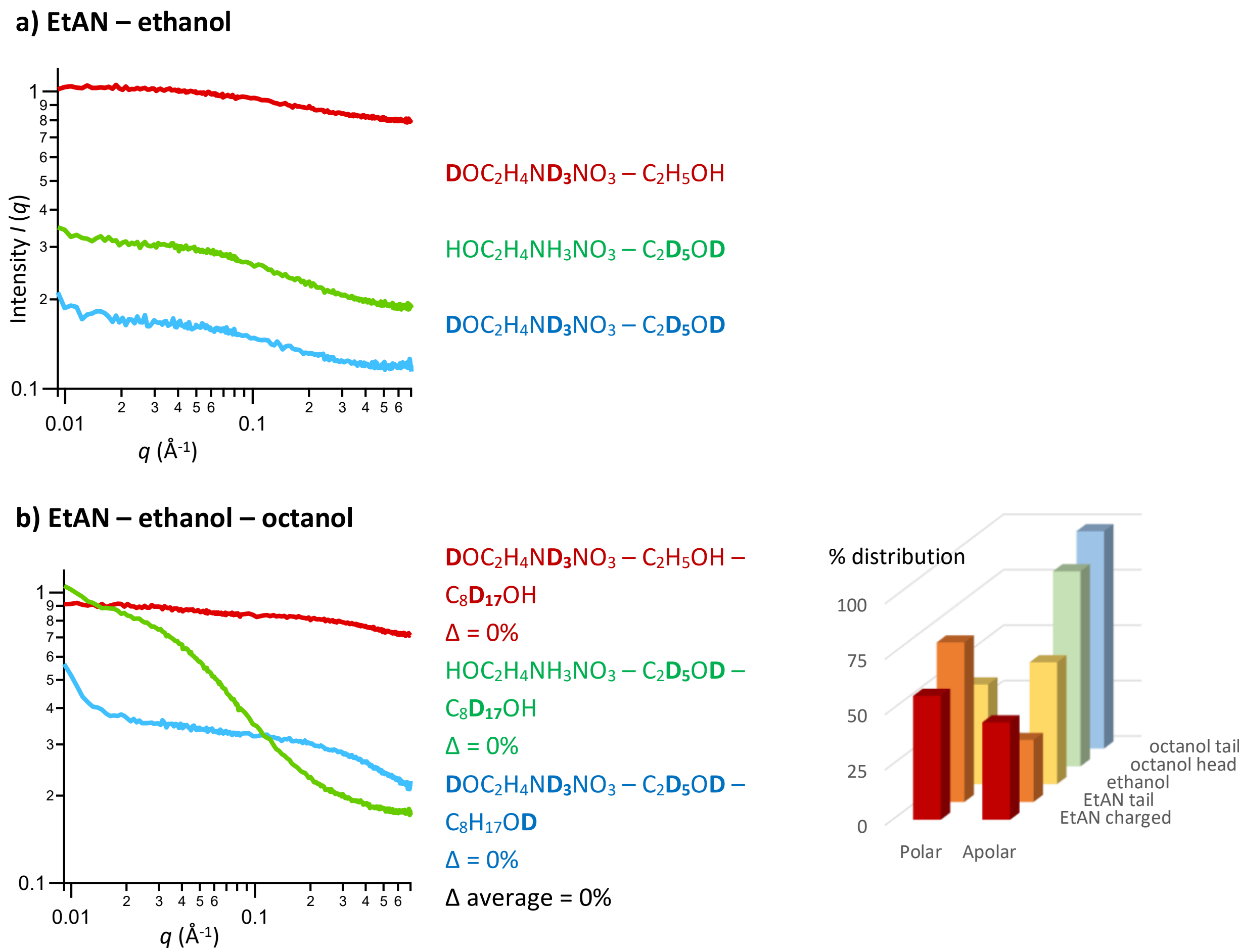

Figure S7 EtAN–ethanol with an additional apolar component; SANS patterns with multiple contrasts at 298K (labels shown in colour), their corresponding Δ (% difference between experimental and theoretical invariant), and the distribution of species across domains. a) EtAN–ethanol (1:2), and b)EtAN–ethanol–octanol (30:60:10). Ratios by wt. based on the fully hydrogenous sample. Deuterated samples have the same molar composition.

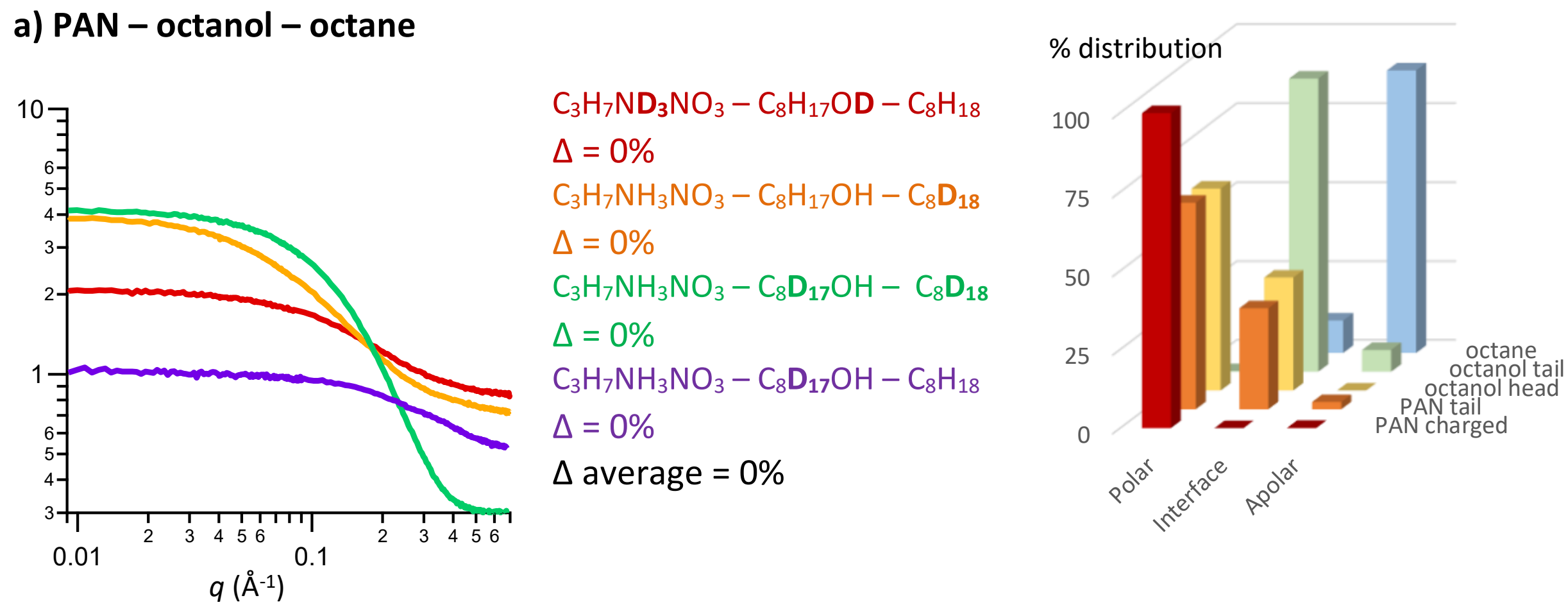

Figure S8 PAN–octanol with an additional apolar component (octane); SANS patterns with multiple contrasts at 298K (labels shown in colour), their corresponding Δ (% difference between experimental and theoretical invariant), and the distribution of species across domains. PAN–octanol–octane (40:40:20) mixture with ratios labelled by wt. based on the fully hydrogenous sample. Deuterated samples have the same molar composition.

Similarly, the inclusion of an apolar component like octane significantly alters the liquid nanostructure. For the PAN–octanol–octane apolar system (Figure S8), an initial two-domain model yields an unacceptably high average Δ of 53%. Upgrading to a three-domain model drops the error to near 0%. The invariant analysis reveals three distinct structural regions: a polar domain containing PAN and octanol headgroups, an apolar domain consisting almost entirely of octane molecules, and an interfacial domain dominated by octanol tails. This confirms that PAN supports the nanoscopic segregation of octanol and octane, with the amphiphilic octanol molecules residing predominantly at the interface to solubilize the non-polar oil.

E. Neutron Diffraction, EPSR Simulation, and Cluster Analysis

Neutron diffraction combined with Empirical Potential Structural Refinement (EPSR) simulation technique is used to rationalize how protic IL-supported self-assembly can be controlled over solute polarity, packing geometry, and formulation at the atomic and nanoscale. EPSR performs a Monte Carlo simulation governed by Lennard-Jones potentials, atom-centered point charges, as well as chemical and physical constraints such as molecular structure and liquid density.

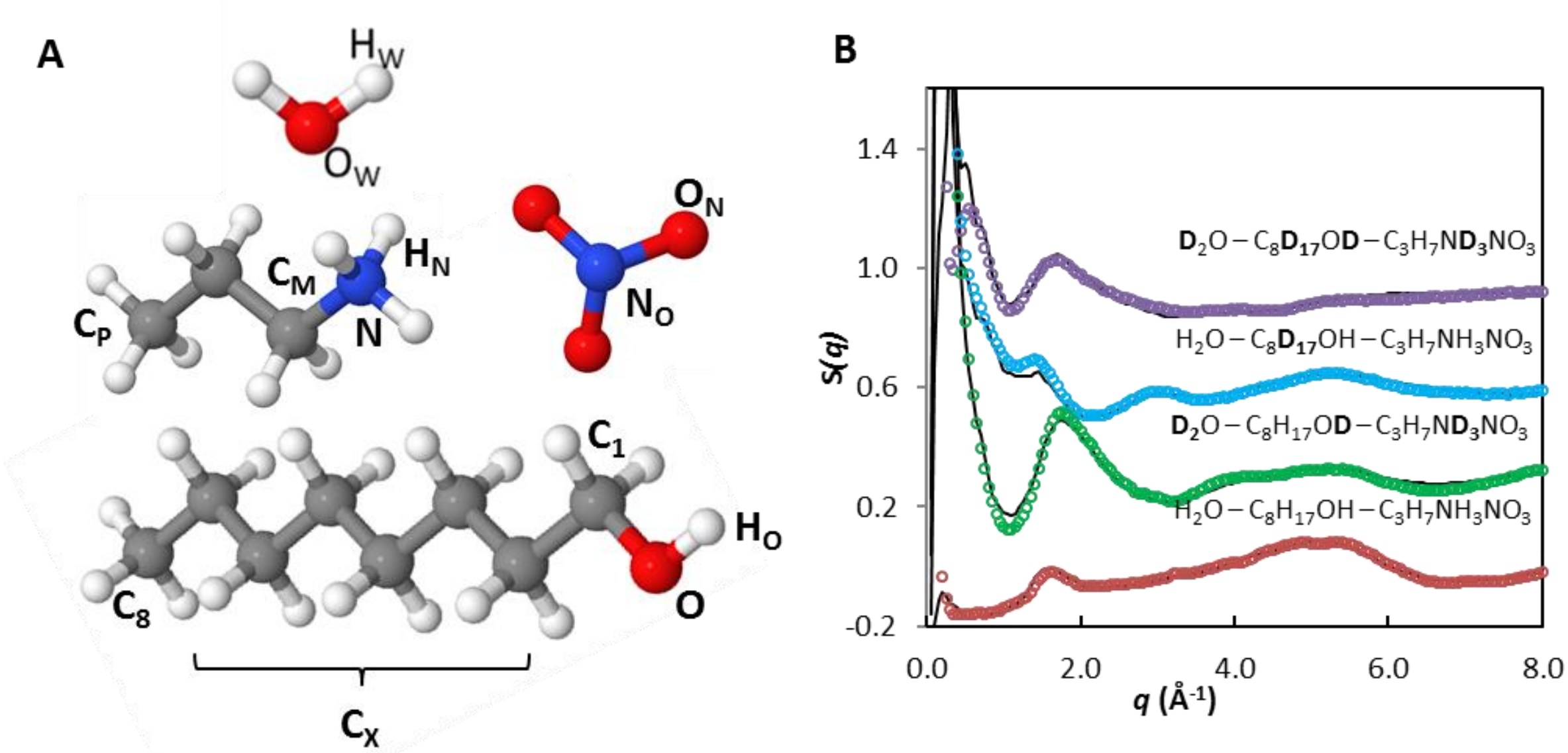

Figure S9 a) Components of the water–PAN–octanol system with atomic labels. Hydrocarbon hydrogens are rendered for clarity. b) Neutron diffraction (coloured data points) and EPSR fitted (black lines) structure factors as a function of the scattering vector, *q* (Å^{-1}), at 298 K. Data are offset for clarity.

For the water-PAN-octanol system (mass ratio 20:60:20), the EPSR simulation box is composed of 2532 water molecules, 1117 PAN ion pairs, and 350 n-octanol molecules. This is an ensemble equivalent to 3.7 x 10^4 atoms, converging in a cubic box of length 72 Å in real space. EPSR simultaneously fits four isotopic substitutions of this mixture at 298K. As shown in Figure S9b, there is an excellent agreement between the measured neutron diffraction data and the EPSR fits across the q-range of interest, demonstrating the consistency of the EPSR model with the measured static structure factor S(q). A signature peak shifts to below 0.3Å-1 upon addition of water, consistent with our SANS studies.

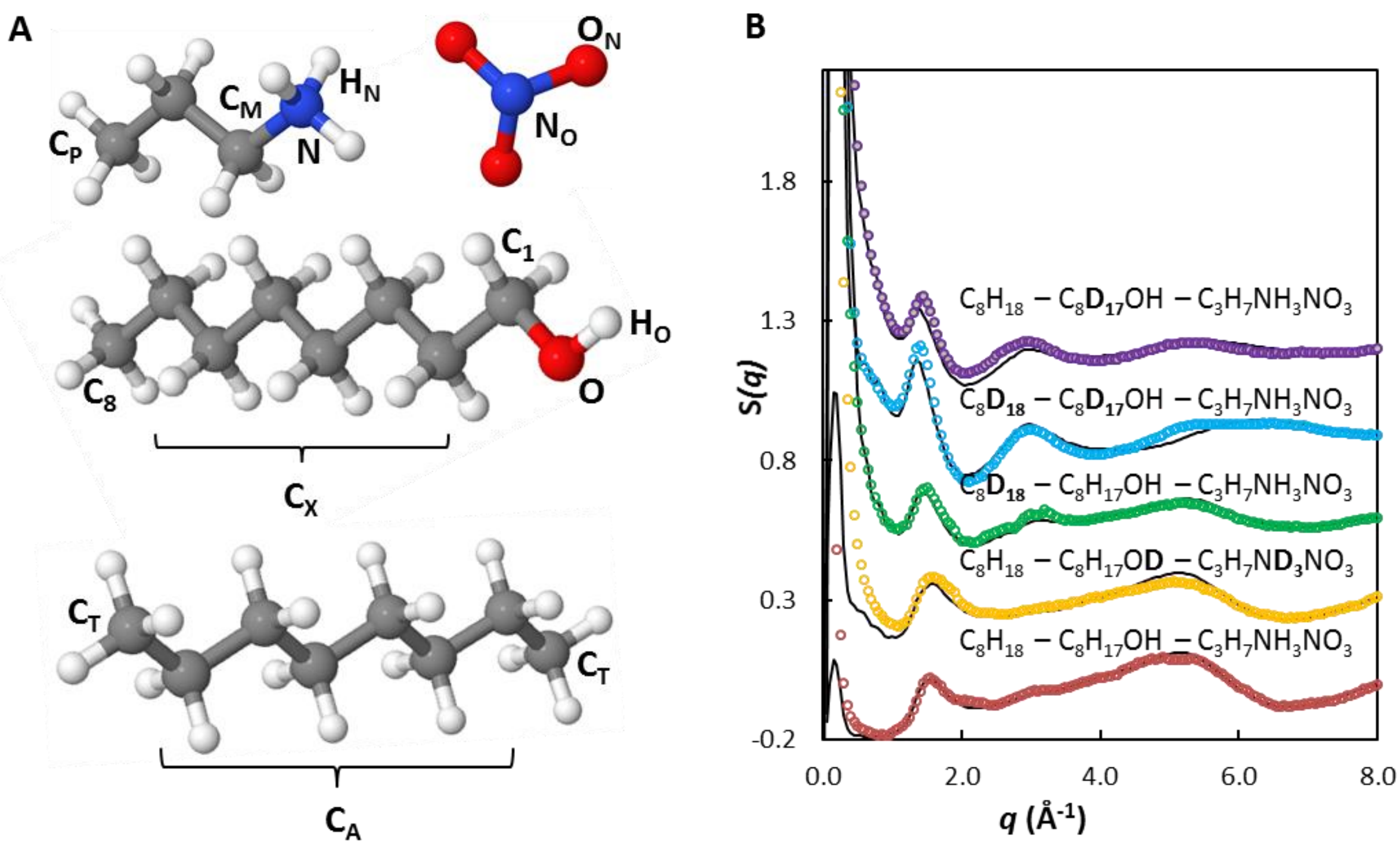

Figure S10 a) Components of the PAN–octanol–octane system with atomic notions used throughout this thesis. Hydrocarbon hydrogens are rendered for clarity. b) Neutron diffraction (coloured data points) and EPSR fitted (black lines) structure factors as a function of the scattering vector, q (Å^{-1}), at 298 K. Data are offset for clarity.

By comparison, the PAN-octanol-octane 'apolar' system (mass ratio 40:40:40 for fully hydrogenous) utilized a simulation box containing 607 PAN ion pairs, 570 octanol molecules, and 324 octane molecules (equivalent to 3.5×10^4 atoms in a 69 Å cubic box). Again, there is excellent agreement for all four isotopic substitutions demonstrating consistency, as shown in Figure S10b.

Cluster analysis tracks the fraction of species in clusters of different sizes (n) present in EPSR-converged ensembles, comparing them to the theoretical 3D percolation threshold ($p(n) = n^{-2.2}$). Finite clusters representing random association are distinguished from continuous, box-spanning networks indicative of bicontinuous nanostructures. Deviations above this threshold indicate cooperativity of association into either finite or continuous structures up to the number of units in the box.

Looking at water clusters as an example, one could examine the distribution of cluster size by defining the distance between two oxygen atoms. To demonstrate the methodology of cluster analysis using an independent system based on literature, Figure S11A shows water-glycerol mixtures at different concentrations, the size distribution of water clusters and glycerol clusters.[21] Simulation snapshots, shown in Figure S11, helps to visualise the distribution of glycerol (in grey) and water (in red) respectively, where X_g represents the fraction of glycerol. Figure S11B shows that percolating water clusters appear (shown by red data points to the right of the blue percolation threshold) in all concentrations apart from $X_g = 0.80$. Similarly, percolating glycerol clusters are found at all concentrations of $X_g \geq 0.25$ (shown by black data points to the right of the blue percolation threshold). Therefore, there is a bipercolating mixture at $0.25 < X_g < 0.50$ (both red and black data points to the right of the blue line).

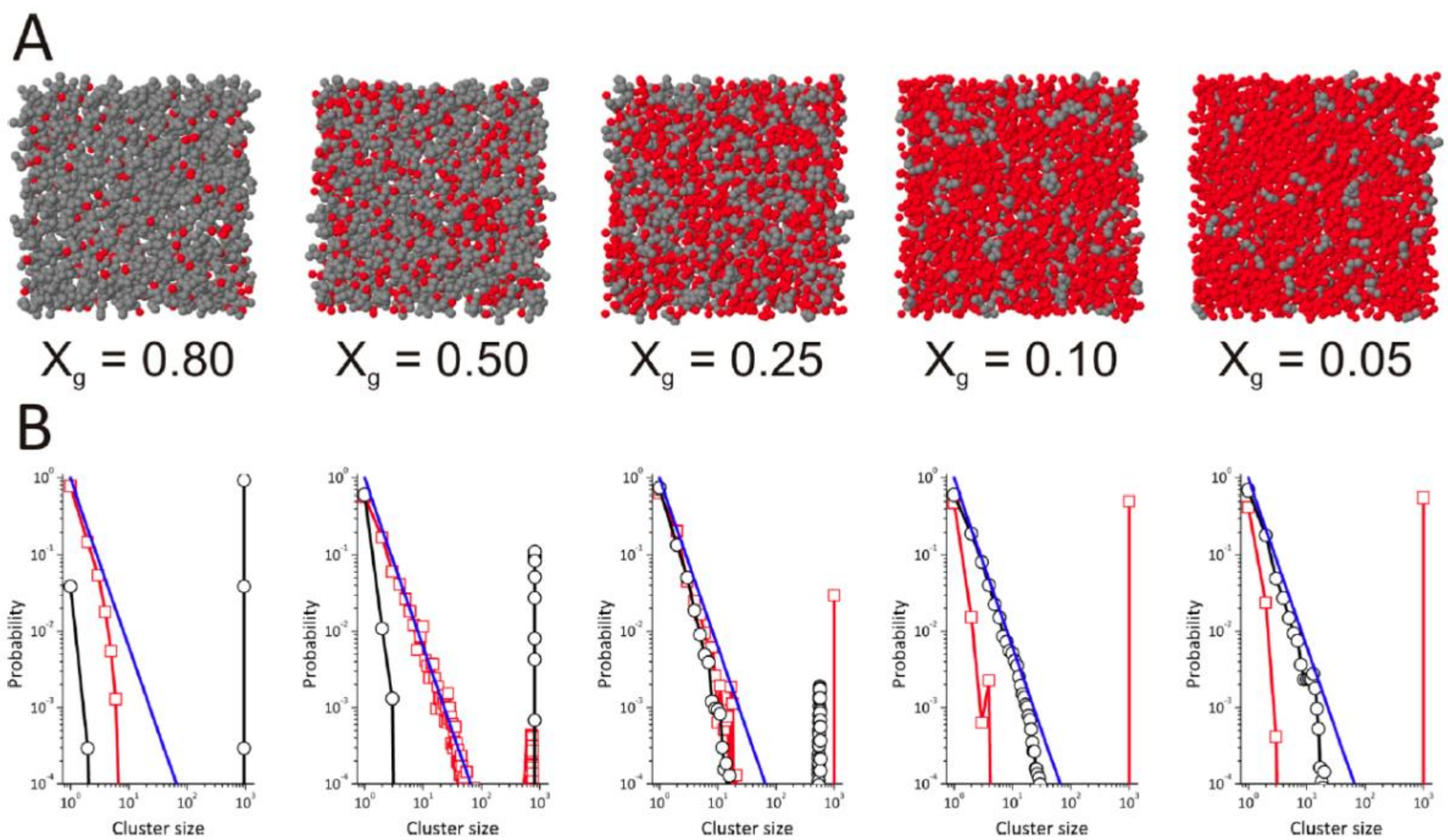

Figure S11. Cluster analysis of water-glycerol mixtures, taken from reference[21] with permission. © 2018 American Chemical Society. A) Experimentally refined simulation snapshots show only glycerol-carbon (grey) and water-oxygen atoms (red) at different concentrations (where X_g is the fraction of glycerol). B) Cluster size distributions for water (red) and glycerol (black), compared to the 3D percolation threshold, $p(n) = n^{-2.2}$ (blue).

SI References

(1) Evans, D. F.; Yamauchi, A.; Roman, R.; Casassa, E. Z. Micelle Formation In Ethylammonium Nitrate, A Low-Melting Fused Salt. *J Colloid Interf Sci* **1982**, *88* (1), 89-96. DOI: 10.1016/0021-9797(82)90157-6.

(2) Gilbert, E. P.; Schulz, J. C.; Noakes, T. J. 'Quokka' - the small-angle neutron scattering instrument at OPAL. *Physica B* **2006**, *385-86*, 1180-1182, Article; Proceedings Paper. DOI: 10.1016/j.physb.2006.05.385.

(3) Wood, K.; Mata, J. P.; Garvey, C. J.; Wu, C.-M.; Hamilton, W. A.; Abbeywick, P.; Bartlett, D.; Bartsch, F.; Baxter, P.; Booth, N.; et al. QUOKKA, the pinhole small-angle neutron scattering instrument at the OPAL Research Reactor, Australia: design, performance, operation and scientific highlightsThis article will form part of a virtual special issue on advanced neutron scattering instrumentation, marking the 50th anniversary of the journal. *Journal of Applied Crystallography* **2018**, *51* (2), 294-314. DOI: doi:10.1107/S1600576718002534.

(4) Soper, A. K.; Howells, W. S.; Hannon, A. C. *ATLAS : analysis of time-of-flight diffraction data from liquid and amorphous samples*; Rutherford Appleton Laboratory, 1989.

(5) Soper, A. K. Joint structure refinement of x-ray and neutron diffraction data on disordered materials: application to liquid water. *J. Phys.-Condes. Matter* **2007**, *19* (33), 18, Article; Proceedings Paper. DOI: 10.1088/0953-8984/19/33/335206.

(6) Soper, A. K. Empirical potential Monte Carlo simulation of fluid structure. *Chem. Phys.* **1996**, *202* (2-3), 295-306. DOI: Doi 10.1016/0301-0104(95)00357-6.

(7) Soper, A. K. Partial structure factors from disordered materials diffraction data: An approach using empirical potential structure refinement. *Phys. Rev. B* **2005**, *72* (10). DOI: ARTN 104204 10.1103/PhysRevB.72.104204.

(8) Soper, A. K. Tests of the empirical potential structure refinement method and a new method of application to neutron diffraction data on water. *Molecular Physics* **2001**, *99* (17), 1503-1516. DOI: 10.1080/00268970110056889.

(9) Diat, O. K., M. L. ; Touraud, D.; Deme, B. ; Grillo, I.; Kunz, W.; Zemb T. Octanol-rich and water-rich domains in dynamic equilibrium in the pre-ouzo region of ternary systems containing a hydrotrope. *Journal of Applied Crystallography* **2013**, *46*, 1665-1669. DOI: 10.1107/S002188981302606X.

(10) Moriyoshi, T.; Sakamoto, T.; Uosaki, Y. (liquid+liquid) Equilibria of (water plus ethanol plus a C8 alkanol) from 0.1 to 200 mpa at 298.15K. *J. Chem. Thermodyn.* **1989**, *21* (9), 947-954, Article. DOI: 10.1016/0021-9614(89)90153-5.

(11) Zana, R. Aqueous surfactant-alcohol systems - a review. *Adv Colloid Interfac* **1995**, *57*, 1-64, Review. DOI: 10.1016/0001-8686(95)00235-i.

(12) Eastoe, J.; Hatzopoulos, M. H.; Dowding, P. J. Action of hydrotropes and alkyl-hydrotropes. *Soft Matter* **2011**, *7* (13), 5917-5925, Review. DOI: 10.1039/c1sm05138e.

(13) Jiang, H. J.; FitzGerald, P. A.; Dolan, A.; Atkin, R.; Warr, G. G. Amphiphilic self-assembly of alkanols in protic ionic liquids. *The journal of physical chemistry. B* **2014**, *118* (33), 9983-9990. DOI: 10.1021/jp504998t.

(14) Atkin, R.; Warr, G. G. The smallest amphiphiles: Nanostructure in protic room-temperature ionic liquids with short alkyl groups. *J. Phys. Chem. B* **2008**, *112* (14), 4164-4166. DOI: 10.1021/jp801190u.

(15) Edward, J. T.; Farrell, P. G.; Shahidi, F. PARTIAL MOLAL VOLUMES OF ORGANIC-COMPOUNDS IN CARBON-TETRACHLORIDE. 1. ALKANES - CONFORMATIONAL EFFECTS. *J. Phys. Chem.* **1978**, *82* (21), 2310-2313, Article. DOI: 10.1021/j100510a012.

(16) Gruen, D. W. R. A model for the chains in amphiphilic aggregates. 1. Comparison with a molecular dynamics simulation of a bilayer. *The Journal of Physical Chemistry* **1985**, *89* (1), 146-153. DOI: 10.1021/j100247a032.

(17) http://www.ncnr.nist.gov/resources/activation/

(18) Porod, G. Die Rontgenkleinwinkelstreuung Von Dichtgepackten Kolloiden Systemen .1. *Kolloid-Zeitschrift and Zeitschrift Fur Polymere* **1951**, *124* (2), 83-114, Article. DOI: 10.1007/bf01512792.
(19) Porod, G. Die Rontgenkleinwinkelstreuung Von Dichtgepakten Kolloiden Systemen .2. *Kolloid-Zeitschrift and Zeitschrift Fur Polymere* **1952**, *125* (1), 51-57, Article. DOI: 10.1007/bf01519615.
(20) de Campo, L.; Varslot, T.; Moghaddam, M. J.; Kirkensgaard, J. J. K.; Mortensen, K.; Hyde, S. T. A novel lyotropic liquid crystal formed by triphilic star-polyphiles: hydrophilic/oleophilic/fluorophilic rods arranged in a 12.6.4. tiling. *Phys. Chem. Chem. Phys.* **2011**, *13* (8), 3139-3152, Article. DOI: 10.1039/c0cp01201g.
(21) Towey, J. J.; Soper, A. K.; Dougan, L. Molecular insight into the hydrogen bonding and micro-segregation of a cryoprotectant molecule. *The journal of physical chemistry. B* **2012**, *116* (47), 13898-13904. DOI: 10.1021/jp3093034.
